# Increasing Rumen Microbial Diversity in Goats Favours the Adaptation to High‐Concentrate Diets With Minor Effects on Feed Utilization

**DOI:** 10.1111/jpn.70024

**Published:** 2025-10-28

**Authors:** Alejandro Belanche, Juan Manuel Palma‐Hidalgo, David R. Yáñez‐Ruiz

**Affiliations:** ^1^ Estación Experimental del Zaidín (CSIC) Granada Spain; ^2^ Departamento de Producción Animal y Ciencia de los Alimentos Universidad de Zaragoza Zaragoza Spain

**Keywords:** anaerobic fungi, caecal microbiota, feed utilization, gene expression, methanogens, protozoa, ruminants

## Abstract

Evolution has enabled ruminants to develop a complex rumen microbiota that aids in the digestion of fibrous feeds. This study examines whether promoting a highly diverse rumen microbiota during early life continues to offer long‐term benefits in modern dairy production systems, where young ruminants are reared without exposure to adult ruminants and are fed high‐concentrate diets. A total of 36 newborn goat kids were divided in 4 groups. During the first 10 weeks of age, animals were daily inoculated with autoclaved rumen fluid (AUT), fresh rumen fluid from adult goats fed forage (RFF), or concentrate diet (RFC), or received no inoculation (CTL). At 29 weeks of age, following an 18‐week wash out period, the animals were shifted from a full‐forage to a high‐concentrate diet to assess their ability to adapt and digest this later diet. Results revealed that early life inoculation with fresh rumen fluid had a lasting effect on the rumen microbiota, promoting higher bacterial (+93 OTUs), methanogens (+5 OTUs) and protozoal diversity (+23 OTUs), whereas CTL animals remained protozoa‐free. This superior microbial complexity accelerated the adaptation to high‐concentrate diets, decreased digestive disorders (rumen acidosis and diarrhoea) and increased BW gain. Once adapted to the diet, inoculated animals exhibited higher rumen VFA concentration (+16%), blood glucose (+28%), rumen papillae width (+43%) and increased expression of rumen epithelium genes involved in the cell proliferation (Cyclin 1), VFA absorption (MCCT1) and VFA metabolism (HMGCL), suggesting an enhanced energy uptake capacity. Inoculation with autoclaved rumen fluid as source of VFA had lower long‐term effects compared to fresh inocula. No differences across treatments were noted for feed digestibility, N excretion, and microbial protein synthesis. In conclusion, promoting greater rumen microbial diversity is a desirable strategy to prevent digestive disorders during the adaptation process to high‐concentrate diets, having minor effects once the animals are adapted to this diet.

**Abbreviations**
ADF: acid detergent fibre
CP: crude protein
DM: dry matter
EE: ether extract
N: nitrogen
NDF: neutral detergent fibre
OTU: operational taxonomic unit
PCoA: principal coordinate analysis
PD: purine derivatives
VFA: volatile fatty acids

## 1 Background

Evolution have conferred ruminants several digestive adaptations. They involve the development of a complex, multi‐chambered forestomach, a system of regurgitation and rumination and the presence of a highly diverse rumen microbiota composed of bacteria, methanogenic archaea, protozoa, anaerobic fungi and phages (Youngblut et al. 2019). This rumen microbial fermentation provides several competitive advantages, such as improved fibre digestion and microbial protein synthesis, but also some drawbacks including the energy lost in form of enteric CH_4_ emissions and the low efficiency of feed utilization (Huws et al. 2018). In light of these considerations, it is widely accepted that a highly diverse rumen microbiota offers distinct advantages to ruminants, especially when they are primarily fed forage diets (Weimer et al. 2009). However, it is still unknown if promoting a highly diverse rumen microbiota could provide similar advantages when ruminants are fed high‐concentrate diets. A study using dairy cows indicated that high efficiency was associated with lower rumen bacterial richness and CH_4_ emissions in cows fed concentrate diets (Shabat et al. 2016). Another study reported no differences in rumen bacterial diversity between feed efficient and non‐feed efficient steers when fed a high concentrate diet made of 57% rolled corn, 30% distillers grains, 8% alfalfa hay and 5% of supplements (Myer et al. 2015). Similarly, no differences in the bacterial and fungal diversities, but higher methanogens diversity and Bacteroidetes to Firmicutes ratio were observed in steers fed mixed diets (Lopes et al. 2021). The variability in outcomes across these studies highlights the critical role of diet composition. It suggests that there may not be a universal ‘one‐size‐fits‐all’ approach to optimizing rumen function across diverse ruminant production systems. Instead, the interplay between diet and the rumen microbiota appears to be a complex and context‐dependent phenomenon, deserving further exploration and tailored strategies to enhance ruminant productivity.

In the context of feeding high yielding ruminants, the market favours high‐concentrate diets based on cereal grains as they represent a lower cost per Mcal of Net Energy. On the contrary, high‐quality forages are often scarce in many regions worldwide, have high transportation costs, and have handling challenges which incentive the reduction of forage inclusion to enhance operational efficiency in intensive dairy farms. In this sense, most dairy ruminants are typically fed high‐forage diets during the dry period to prevent excessive fat deposition. However, after parturition, they are rapidly transitioned to high‐concentrate diets to meet their increased nutrient requirements. It has been observed that an abrupt shift from ad libitum access to a forage diet to a high‐concentrate diet can trigger metabolic disorders in the host, including rumen acidosis, diarrhoea, weight loss, and reduced productivity (Owens et al. 1998). Brown et al. (2006) demonstrated that this adaptation is highly variable across animals, moreover the animals that better coped with this adaptation where those which maintain the protozoal population and had increased levels of lactate‐utilizing bacteria. Hence, there is a growing interest in modulating the rumen microbial community to enhance ruminant resilience to dietary changes.

Modern intensive dairy systems are particularly susceptible to these digestive disorders because newborns are often separated from their mothers and artificially reared with milk replacers. This separation leads to a delay in the rumen microbial and physiological development and an absence of rumen protozoa that can persist as long as the animals are not in contact with adult ruminants (Belanche et al. 2011, 2019d; Palma‐Hidalgo, Yáñez‐Ruiz et al. 2021). Early‐life nutritional interventions have been proposed to influence rumen microbial colonization, leading to short and potentially long‐term effects on the rumen microbial community structure and animal productivity (Yáñez‐Ruiz et al. 2015).

Supplementation of young calves with sodium butyrate has been associated to greater rumen anatomical development when mixed with the starter feed, whereas a modulation of the rumen microbiota (O'Hara et al. 2018), as well as greater improvements in rumen development, health and BW gain were reported when sodium butyrate was mixed with the milk replacer (Górka et al. 2018). Several authors have investigated with different degrees of success the effects of inoculating young ruminant with cell‐free rumen fluid, bacterial polysaccharides and lyophilized rumen fluid (Muscato et al. 2002; Zhong et al. 2014). In companion publications from the present experiment, we demonstrated that the inoculation of young ruminants with rumen fluid from adult ruminants (Belanche et al. 2020), or even the presence of adult companions (Palma‐Hidalgo, Yáñez‐Ruiz et al. 2021), represent successful strategies to accelerate the rumen microbial and functional development. Both strategies allowed to optimize the weaning process for artificially reared ruminants, as well as the use of forage diets (Belanche, Palma‐Hidalgo et al. 2023). However, the persistency of these effects in later life and the potential advantages during the adaptation and utilization of concentrate‐diets remain unknown.

The objective of this study was to elucidate the long‐term benefits and drawbacks of promoting a highly diverse rumen microbiota through early‐in‐life inoculation with rumen fluid from adult ruminants for optimizing intensive ruminant production systems based on high‐concentrate diets. This study encompassed a multi‐kingdom analysis of the rumen microbiota (including bacteria, protozoa, methanogens and anaerobic fungi), an examination of the hindgut microbiota, and gene expression in the gastro‐intestinal tract (GIT). Special attention was given to understand the effect of these interventions on the adaption process to high‐concentrate diets and their implication for feed utilization, productivity and overall animal health.

## 2 Methods

### 2.1 Inocula Preparation and Inoculation

**Figure 1:**
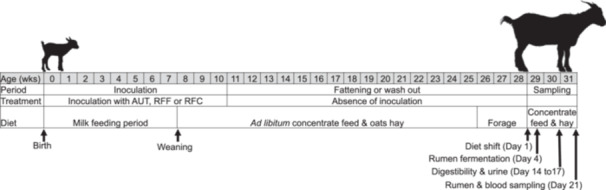
Illustration of the different stages of the experimental set up.

A total of 36 Murciano‐Granadina goat kids, aged 2 days, were divided randomly into four experimental groups (*n *= 9). These groups were kept physically separated throughout the entire experiment to prevent direct contact between animals from different treatments. The experiment consisted in three periods as follows (Figure 1): (1) the inoculation period from birth to Week 10 which included artificial milk feeding for 7 weeks plus the post‐weaning phase; (2) the wash out period (from Week 10 to 28 of age) in which the animals did not receive any further inoculation and were fed a fattening diet (up to Week 25) followed by a forage diet (Week 26–28 of age); and (3) the sampling period (from Week 29 to 31 of age) to evaluate the long‐term effects of the inoculation after the animals were shifted to a high‐concentrated diet.

The treatments included oral inoculation from Week 1 to 10 of age with autoclaved rumen fluid (AUT), rumen fluid adapted to a forage (RFF), rumen fluid adapted to concentrate diet (RFC) or absence of inoculation (CTL). Inoculation was performed daily to encourage distinct development in the rumen microbiota, as previously described (Belanche et al. 2020; Palma‐Hidalgo, Jiménez et al. 2021). The rumen inocula were obtained from eight adult goats with rumen fistulas that were fed either a forage diet (oats hay, *n* = 4) or a high‐concentrate diet (75% concentrate feed and 25% oats hay, *n* = 4). Rumen fluids were collected 2 h after the morning feeding, pooled by diet type, filtered through cheesecloth, and orally inoculated as fresh inoculum to the young ruminants (2.5 mL/animal during week 1 and 5 mL/animal thereafter). The autoclaved inoculum was prepared weekly by mixing equal volumes of autoclaved RFF and RFC inocula. This mixture was autoclaved at 115°C during 30 min to lyse all microbes while preserving similar concentrations of fermentation products. All inocula were sampled for microbial characterization.

All goat kids were initially fed commercial milk replacer (Univet Spray, Cargill, Minnesota, USA), weaned at 7 weeks of age and raised until 31 weeks of age. From Week 2 to 25 of age, all animals had unlimited access to pelleted concentrate (0–14 Rumiantes Transición, Macob, Granada, Spain) and oats hay. From week 26 to week 28 animals were fed only forage (oats hay). When animals reached 28 weeks of age, they were subjected to a nutritional challenge involving an abrupt dietary shift to assess adaptability to a high‐concentrate diet (Day 1). For the following 3 weeks, all animals received a high‐concentrate diet consisting of concentrate feed [0–14 Rumiantes Transición, Macob, Granada, Spain, chemical composition in g/kg dry matter (DM): 938 organic matter (OM), 199 crude protein (CP), 323 neutral detergent fibre (NDF), 132 acid detergent fibre (ADF), 15 acid detergent lignin (ADL)] and oats hay (930 OM, 79 CP, 634 NDF, 280 ADF, 55 ADL), both offered ad libitum.

### 2.2 Rumen and Blood Sampling, Performance and Health

To assess the adaptation of the animals to the concentrate diet, they were housed in individual pens (2 × 2 m) and feed intake, BW change and presence of diarrhoea was daily monitored during 21 days after the dietary change. Average DMI and BW change was calculated per animal during the beginning (Day 1–4) and the end (Day 18–21) of the experimental period to assess the speed of adaptation to the high‐concentrate diet. Additionally, on the fourth day after the shift to this new diet, the rumen fermentation was assessed as previously reported (Palma‐Hidalgo, Jiménez et al. 2021). In brief, approximately 50 mL of rumen content was obtained through oro‐gastric intubation at 9:00 h, filtered using cheesecloth, and pH was immediately measured. Four samples were taken for the analysis of volatile fatty acids (VFA), ammonia, lactate, and microbial DNA extraction (Belanche, Yáñez‐Ruiz et al. 2019).

On Day 21, blood (4 mL) was sampled from the jugular vein, placed in tubes without anticoagulant for 20 min at room temperature and centrifuged at 2000*g* for 15 min. Serum was extracted and stored at −80°C. This serum was further used to determine the concentrations of glucose, β‐hydroxybutyrate, blood pH, pCO2, tCO2, Na+, K+, HCO3−, Cl−, and anion gap were determined using an auto‐analyzer (BA400, Bio‐Systems, Barcelona, Spain). Additionally, cortisol concentration in the hair was used as an indicator of the stress (Belanche et al. 2020). A surface of 25 cm^2^ in the neck was shaved both before (Day 1) and 21 days after the dietary shift to collect the hair that grew during this specific period. Cortisol concentration was measured using a commercial kit (Cortisol ELISA Saliva, ALPCO, Salem, NH).

### 2.3 Digestibility and Microbial Protein Synthesis

Following 14 days of adaptation to the concentrated diet, the goats were placed in metabolic crates for a 4‐day period (Days 14–17) to assess feed digestibility (Belanche et al. 2011) in two consecutive periods. Feed intake, faecal and urinary excretion were daily monitored and pooled to determine feed digestibility and urinary purine derivative (PD). Dry matter (DM) and organic matter (OM) concentrations were determined using the method 934.01, 942.05, respectively (AOAC 2005). Nitrogen (N) content was also measured using the methods 990.03 and 993.13 for solid and liquid samples, respectively. Neutral detergent fibre (NDF) and acid detergent fibre (ADF) were measured (Van Soest et al. 1991) using an Ankom 220 fibre analyser unit (Ankom Technology Crop., Macedon, NY) with α‐amilase and expressed without residual ash. The urine concentration of purine derivatives (PD) and creatinine were determined using a HPLC system (Balcells et al. 1992) using Allopurinol as internal standard. The urinary PD excretion divided by the intake of digestible OM (DOMI) during the digestibility assessment was used as an indicator of the efficiency of microbial protein synthesis.

### 2.4 Gut Anatomical Development and Gene Expression

At Day 21 after the dietary shift all animals were euthanized, gut was dissected and the weight of the different gut compartments was recorded, including the full sections of the GIT (rumen, omasum, abomasum, small intestine and large intestine) and the empty rumen. Two samples of rumen epithelium were taken from the cranial ventral sac: one sample (5 × 5 cm) was fixed with formaldehyde (4%) for subsequent histological examination, while the other sample (0.5 × 0.5 cm) was rinsed with PBS buffer and snap frozen for RNA extraction. Rumen content was also collected as previously described for pH, ammonia, lactate, VFA and microbial DNA extraction. Additionally, rumen liquid (12.5 mL) was mixed with 37.5 mL of anaerobic buffer and incubated at 39°C for 24 h in 120‐mL Wheaton bottles to determine in vitro gas and methane (CH_4_) production (Belanche et al. 2015). Caecum content was also sampled, mixed 1:1 ratio with PBS buffer and filtered. This allowed to determine the caecum pH, VFA concentration and microbial DNA extraction to assess the impact of the dietary treatment on the hindgut microbial fermentation.

Rumen epithelia was cut in slices (1 mm wide) in triplicate and a photo was taken using optical stereomicroscope (M165FC Leica Microsystems, Wetzlar, Germany). Measurements of the rumen papillae length and width were determined using the Leica Application Suite X Life Science software. Gene expression of targeted genes from the rumen and caecal epithelium was assessed to determine the potential impact of the nutritional intervention on the gut anatomical development as previously reported (Belanche, Arturo‐Schaan et al. 2023). Briefly, frozen epithelial samples (100 mg FM) were physically disrupted using a Bullet Blender homogenizer (Next advance Inc. New York, NY) and RNA was extracted using the TRIzol Reagent (Invitrogen, Carlsbad, CA, Abecia et al. 2017). Extracted RNA (1 µg) was reverse‐transcribed (QuantiTect Revese Transciption kit, Qiagen, UK) to determine the expression of genes related to: (i) VFA absorption such as monocarboxylate transporter isoform 1 (*MCT1*) and putative transporter isoform 1 (*PAT1*); (ii) VFA metabolism such as β‐hydroxybutyrate dehydrogenase isoform 1 (*BHD1*), 3‐hydroxy‐3‐methylglutaryl‐CoA synthase (*HMGCS1*) and 3‐hydroxy‐3‐methylglutaryl‐CoA lyase (*HMGCL*); (iii) cell proliferation such as *Cyclin D1*, *Cyclin A* and cycling‐dependent kinase isoform 2 (*CDK2*); (iv) epithelial growth such as insulin growth factor isoform 1 *(IGF1*) and IGF1 receptor (*IGF1R*) and (iv) apoptosis such as B‐cell lymphoma (*BCL‐2*) and BCL‐2 associated X protein (*BAX*) and Caspase‐3. Quantitative PCR was performed using a SYBR Green (Sigma Aldrich, Madrid, Spain) on a Bio‐Rad iCycler (Bio‐Rad Kaboratories LTD., Mississauga, ON). Primers and qPCR conditions are described in Table S1. The relative expression of each gene was normalized to the *β‐actin* as housekeeping gene, and the data were calculated by the $2^{{–\Delta \Delta C}_{T}}$ method (Kenneth and Schmittgen 2001).

### 2.5 Gut Microbiota

To characterize the rumen and caecal microbial composition, we followed a systematic procedure (Palma‐Hidalgo, Jiménez et al. 2021). Briefly, frozen rumen samples were freeze‐dried and then subjected to physical disruption by bead‐beating for a duration of 1 min. Subsequently, DNA was extracted using a commercial kit (QIAamp DNA Stool Mini Kit, Qiagen LTd., Barcelona, Spain). DNA extractions were conducted for rumen and caecal samples, rumen fluids used as inocula, as well as negative controls (DNA extraction without rumen fluid) to ensure the reliability of the results.

Gut concentration of the different microbial groups were determined by qPCR using serial dilutions of microbial standards (Belanche, Cooke et al. 2019) and specific primers for the 16S rRNA gene for bacteria, the mcrA gene for methanogens and 18S rRNA genes for protozoal and anaerobic fungi (Table S1). For meta‐taxonomic analyses, DNA samples were sent to University of Illinois Biotechnology Center (Urbana, IL, USA). Amplicon sequencing was performed using Miseq V3 platform (Ilumina Inc., San Diego, CA, USA) and specific primers to amplify bacterial 16S (V3‐V5 region), methanogens 16S, protozoal 18S and anaerobic fungi ITS3‐ITS4 regions (Table S1) as previously described (Palma‐Hidalgo, Jiménez et al. 2021). For each of the four major microbial groups, samples were sorted by barcode, demultiplexed, adapters were removed and paired‐end reads were merged and combined into a unique file. Subsequent data analysis was carried out using QIIME2 (Version 2021.4) and Mothur (Schloss et al. 2009) for archaea (Caporaso et al. 2010), PIPITS for fungi (Gweon et al. 2015) and IM‐Tornado for bacteria and protozoa (Jeraldo et al. 2014), where non‐overlaping reads were processed while retaining maximal information. Low‐quality reads (THRED score < Q25) were trimmed and chimeric sequences were eliminated using chimera.vsearch (Edgar et al. 2011) as previously described (Palma‐Hidalgo, Jiménez et al. 2021; Belanche, Palma‐Hidalgo et al. 2023). Sequencing analysis generated 14893 ± 6819, 2131 ± 1446, 17995 ± 5872 and 8059 ± 6304 high quality sequences per sample for bacteria, methanogens, protozoa and anaerobic fungi, respectively. All sequences were then grouped into operational taxonomic units (OTUs) based on a 97% similarity threshold. Taxonomic classification of the resultant OTUs was achieved using Silva_138 database (Quast et al. 2013) for bacteria and protozoa, RIM‐DB for methanogens (Seedorf et al. 2014) and UNITE for fungi (Nilsson et al. 2019). After taxonomical classification, data from each of the four major microbial groups were processed independently. Sequencing depth was normalized to equal sequence counts established by the sample that presented the lowest number of sequences, (7000, 1000, 10000 and 1000 for bacteria, methanogens, protozoa and anaerobic fungi, respectively). Afterwards, singleton OTUs were excluded and the relative abundance of each OTU was determined along with the Good's coverage and alpha diversity indices (Richness, Shannon's and Simpson's indexes).

### 2.6 Calculations and Statistical Analyses

Statistical calculations were performed using SPSS software (IBM Corp., Version 21.0, NY, USA). For rumen fermentation data were analysed based on a repeated measures mixed‐effects (residual maximum likelihood) as follows:

Where *Y*_ijkl_ is the dependent, continuous variable, *µ* is the overall population mean, *I*_*i*_ is the fixed effect fo the inoculation (*I* = CTL vs. AUT vs RFF vs RFC), *T*_*j*_ is the fixed effect of the sampling time (*j* = 4d vs. 21d), *(I x T)*_*ij*_ is the interaction term, *G*_*k*_ is the random effect of the period (*k* = 1 vs. 2), *A(G)*_*l*_ is the random effect of the animal nested to the period (*l* = 1–36), and *e*_ijkl_ is the residual error. A single time point ANOVA (21 days) was used for blood metabolites, digestibility and urinary PD excretion. Fermentable organic matter (FOM) was calculated based on the VFA concentration (Moss et al. 2000). Taxa abundances (in %) were tested for normality using the Shapiro–Wilk test and data were analysed using the Kruskal–Wallis non‐parametric test. The Bonferroni post hoc test was used to minimize the false discovery rate (FDR). Significant effects were declared at *p* < 0.05 and tendency to difference at *p* < 0.1.

Treatment effects on the beta diversity of the bacterial, methanogens, protozoal and anaerobic fungi communities were assessed based on the Bray‐Curtis distance metrics using the UPGMA function (PRIMER‐6 software, PRIMER‐E Ltd., Plymouth, UK). Abundances of each OTU were log10‐transformed and the treatments effects on the microbial community structure were analysed by non‐parametric PERMANOVA after 999 random permutations of residuals under the reduced model using the Montecarlo test. When significances were detected, pair‐wise comparisons were performed across treatments. For each microbial group, a principal Coordinate analysis (PCoA) was conducted to illustrate the impact of the treatments on the overall community structure. Additionally, tripod vectors were included to illustrate the relationships between the community structure and the metadata consisting of 31 variables encompassing rumen fermentation, digestibility, purine derivatives, blood metabolites and microbial diversity. To comprehensively assess the effects of the treatments on the overall rumen microbiome, a multi‐kingdom analysis was performed considering abundances from all microbial groups. To identify associations between microbial taxa abundances and the metadata, Spearman correlations were calculated and strong correlation discussed further (*ρ* ≥ 0.4 or ≤ −0.4 and *p* < 0.001).

## 3 Results

### 3.1 Inoculum and Rumen Fermentation

Differences across rumen inocula used for the inoculation of the experimental animals have already been described in terms of their fermentation pattern and microbiota (Palma‐Hidalgo, Jiménez et al. 2021). In brief, AUT inocula had similar concentration of fermentation products (e.g., ammonia and VFA) than fresh inocula but without viable microbial cells (Table S2). Additionally, the RFC inocula exhibited the highest total VFA concentration and propionate molar proportion, while RFF inocula showed the greatest acetate molar proportion and AUT exhibited intermediate values. On the contrary, RFC inocula displayed a higher Firmicutes/Bacteroidota ratio and had higher concentrations of protozoa and anaerobic fungi, but lower bacterial and methanogens richness (Table S2).

All animals involved in the study underwent an adaptation process as they transitioned from a forage‐based diet to a high‐concentrate diet. This dietary shift was evident in the rumen fermentation data collected at 4 and 21 days after the abrupt dietary shift (Table 1). During this adaptation period, there was a notable increase in DMI (*p* = 0.001), total VFA (*p* = 0.019), lactate (*p* = 0.028), branched chain volatile fatty acids (BCVFA, *p* = 0.086) and protozoal concentration (*p* = 0.028).

**Table 1:** Feed intake and rumen microbial fermentation in 7‐month‐old goats at 4 and 21 days after an abrupt dietary shift from a forage diet to a high‐concentrate diet. Goats received early‐life daily inoculations during their first 10 weeks of age with autoclaved rumen fluid (AUT), rumen fluid from adult animals fed a forage diet (RFF) or concentrate diet (RFC), or no inoculation (CTL).

		Treatment					*p* values		
	Day	CTL	AUT	RFF	RFC	SED	Ino.	Time	IxT
BW change (g/d)	1–4	−31.2	167	40.3	151	81.20	0.072	0.855	0.709
	4–21	−19.2	73.3	92.8	50.6				
DMI (g/d)	1–4	746	732	566	728	158.0	0.533	< 0.001	0.995
	18–21	1024	998	838	1034				
Forage (% in DM)	1–4	17.9^b^	25.4^ab^	32.7^a^	20.6^ab^	3.947	0.208	0.184	0.005
	18–21	26.3^a^	16.3^b^	22.8^ab^	24.0^ab^				
Rumen pH	4	5.94^b^	6.16^ab^	6.56^a^	6.32^a^	0.207	0.122	0.043	0.058
	21	5.87	6.31	5.93	6.07				
Ammonia‐N (mg/dL)	4	13.1	13.1	8.29	13.1	3.356	0.367	0.646	0.014
	21	5.75^b^	14.2^a^	15.5^a^	13.1^a^				
Lactate (mg/L)	4	50.9	103.3	50.5	86.6	22.57	0.111	0.028	0.455
	21	91.5	98.3	84.9	113.7				
Total VFA (mmol/L)	4	90.6	68.8	68.8	79.3	10.08	0.038	0.019	0.178
	21	84.7	75.6	99.0	96.7				
Molar proportion (%)									
Acetate	4	51.0	59.1	53.6	54.3	2.514	0.500	0.314	0.074
	21	55.4	54.5	56.3	54.7				
Propionate	4	38.3	27.0	32.8	33.4	3.358	0.055	0.087	0.336
	21	33.8	30.7	26.7	31.3				
Butyrate	4	7.33	10.1	10.2	9.10	1.817	0.008	0.169	0.706
	21	7.35	10.1	13.4	10.2				
Valerate	4	1.92	1.86	1.50	1.36	0.284	0.901	0.111	0.044
	21	1.60^b^	1.91^ab^	1.82^ab^	2.02^a^				
BCVFA	4	1.42	1.91	1.85	1.82	0.421	0.201	0.086	0.288
	21	1.85	2.73	1.83	1.76				
Rumen concentration^a^									
Bacteria	4	10.4	10.3	10.7	10.8	0.197	0.077	0.086	0.232
	21	10.4	10.2	10.2	10.6				
Methanogens	4	6.04	5.99	6.23	6.47	0.304	0.144	0.581	0.818
	21	6.02	6.01	6.19	6.68				
Protozoa	4	ND	7.57	7.62	8.10	0.475	< 0.001	0.028	0.556
	21	ND	8.01	8.40	8.51				
Anaerobic fungi	4	5.85	6.10	5.66	5.85	0.294	0.911	0.428	0.569
	21	6.01	5.92	5.98	5.81				

*Note:* Means within a row with different superscript differ (*p* < 0.05).

Abbreviations: BCVAFA, branched chain VFA; BW, body weight, DMI, dry matter intake; VFA, volatile fatty acids.

^a^ Rumen concentration in log10(copies/mg DM) based on qPCR data.

Control animals remained protozoa‐free throughout the entire duration of the experiment and confirmed with optical examination in the microscope (Belanche et al. 2010), while the inoculation with fresh rumen fluid promoted the presence rumen protozoa (*p* < 0.001). Animals inoculated with RFF or RFC had a higher butyrate molar proportion (*p* = 0.008), whereas CTL animals tended to have a higher propionate molar proportion (*p* = 0.055) and rumen bacterial concentration (*p* = 0.077). After 21 days of adaptation, all inoculated animals had higher rumen ammonia‐N concentration (interaction, *p* = 0.014), whereas AUT animals had the lowest total VFA concentration (*p* = 0.038). No differences in the rumen concentration of methanogens and anaerobic fungi were detected across treatments.

### 3.2 Health Indicators, Feed Digestibility and Microbial Protein Synthesis

During the 21‐day period following the dietary shift most CTL animals experienced a higher diarrhoea incidence (*p *< 0.001) and prevalence (*p *< 0.001) compared to the RFF and RFC animals (Table 2). Interestingly, animals which were inoculated with RFF or RFC appeared to undergo this adaptation process more rapidly, resulting in lower BW lost during this period (*p* = 0.072). These differences extended to blood metabolite concentration as well. Specifically, inoculation with RFF led to the highest level of blood glucose, β‐hydroxybutyrate, HCO_3_^−^ and anion Gap, while CTL animals had the lowest levels of glucose, HCO_3_^−^ and Anion Gap (*p* < 0.05). These findings may suggest the potential presence of sub‐acute rumen acidosis. No differences in the hair cortisol concentration were noted across treatments.

**Table 2:** Health indicators, blood parameters, feed digestibility, microbial protein synthesis and methane emissions in 7‐month‐old goats at 21 days after an abrupt dietary shift from a forage diet to a high‐concentrate diet. Goats received early‐life daily inoculations during their first 10 weeks of age with autoclaved rumen fluid (AUT), rumen fluid from adult animals fed a forage diet (RFF) or concentrate diet (RFC), or no inoculation (CTL).

	CTL	AUT	RFF	RFC	SED	*p* value
BW (kg)	25.4	25.0	23.4	24.2	1.37	0.552
Diarrhoea incidence (% animals)	83.3^a^	44.4^ab^	11.1^b^	0^b^	16.85	< 0.001
Diarrhoea prevalence (% days)	12.2^a^	6.35^ab^	0.80^b^	0^b^	16.36	< 0.001
Cortisol in hair (ng/mg)	1.50	1.41	1.39	1.33	0.174	0.851
Blood metabolites						
Glucose (mmol/L)	1.61^b^	1.94^ab^	2.22^a^	1.91^ab^	0.192	0.016
β‐hydroxybutyrate (mmol/L)	0.30^a^	0.24^b^	0.30^b^	0.25^a^	0.026	0.022
Blood pH	7.26	7.30	7.36	7.33	0.046	0.209
pCO_2_ (mm of Hg)	62.7	52.6	43.3	49.1	7.300	0.155
tCO_2_ (mm of Hg)	26.9	24.7	23.6	24.6	1.153	0.084
Na^+^ (mmol/L)	25.0	23.0	22.3	23.0	1.020	0.113
K^+^ (mmol/L)	145	145	146	145	1.211	0.854
HCO_3_^−^ (mmol/L)	4.23^c^	4.60^ab^	4.89^a^	4.48^bc^	0.164	0.005
Cl^‐^ (mmol/L)	109	111	111	111	0.966	0.088
Anion gap^a^	14.9^b^	15.5b	17.0^a^	15.2^b^	0.553	0.006
Feed utilization^a^						
DMI (g/d)	704	814	720	743	76.40	0.489
Forage (% in DM)	29.6	18.0	20.1	20.0	6.340	0.277
Apparent digestibility (%)						
DM	75.7	77.7	75.4	74.9	1.930	0.483
OM	77.1	79.0	76.9	76.0	1.851	0.423
N	70.6	77.1	75.9	74.3	4.010	0.325
NDF	66.6	66.0	63.8	60.7	3.220	0.335
ADF	62.7	61.6	59.2	53.8	3.800	0.146
Urinary excretion						
*N* (g/d)	25.8	24.8	29.3	21.1	5.340	0.456
Creatinine (ug/kg MW)	543	591	597	554	101.9	0.964
PD (mmol/d)	10.5	12.5	11.7	11.1	2.211	0.919
PD/Creatinine ratio	1.70	1.80	1.78	1.80	0.118	0.950
PD/DOMI ratio (mmol/kg)	28.7	21.1	22.3	20.9	6.770	0.527
In vitro fermentation^b^						
Gas production (mM/d)	80.3^b^	104^a^	99.2^a^	105^a^	7.960	0.021
Methane (mM/d)	0.96^b^	1.50^a^	1.42^a^	1.51^a^	0.187	0.037
Methane (mM/FOM)	22.7^b^	37.9^a^	25.2^a^	28.0^a^	4.540	0.017

*Note:* Means within a row with different superscript differ (*p* < 0.05).

Abbreviations: ADF, Acid detergent fibre; DM, dry matter; N, nitrogen; NDF, neutral detergent fibre; OM, organic matter; PD, purine derivatives.

^a^ Feed utilization was measured in metabolic crates during 4 consecutive days (Days 14–17) after the dietary shift.

^b^ In vitro fermentation was measured in batch cultures incubated with rumen fluid during 24 h.

Feed digestibility remained consistently high with values exceeding 75% for DM digestibility. However, no differences were noted for DM nor fibre digestibility across treatments. In relation to the *N* metabolism, no differences were noted in *N* digestibility, urinary excretion of PD and creatinine or the PD/DOMI ratio. Contrastingly, the in vitro gas technique revealed higher gas (*p* = 0.0021) and CH4 production (*p* = 0.017) using inoculum from RFF or RFC than from CTL animals.

### 3.3 Rumen Multi‐Kingdom Microbiota

**Figure 2:**
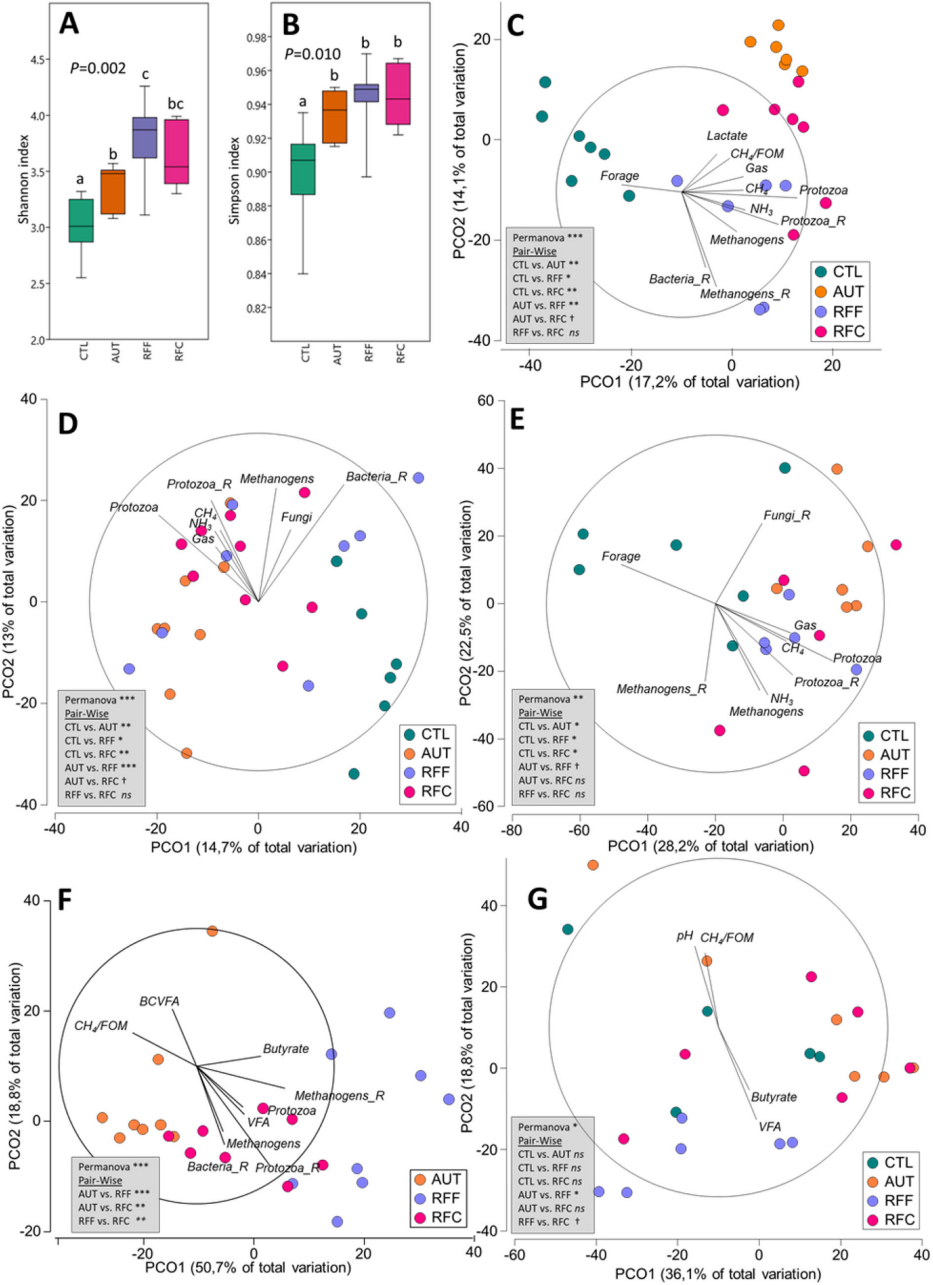
Box plot illustrating the rumen multi‐kingdom diversity in terms of Shannon's (A) and Simpson's index (B) in 7‐month‐old goats at 21 days after an abrupt dietary shift from a forage diet to a high‐concentrate diet. Goats received early‐life daily inoculations during their first 10 weeks of age with autoclaved rumen fluid (AUT), rumen fluid from adult animals fed a forage diet (RFF) or concentrate diet (RFC), or no inoculation (CTL). Principal coordinates analysis illustrating relationships (*ρ* > 0.4) between the structure of the rumen multi‐kingdom microbiota (C), bacteria (D), methanogens (E), protozoa (F) and anaerobic fungi (G) and productive data. PERMANOVA values are provided based on the Bray‐Curtis dissimilarity. BCVFA, branched chain VFA; FOM, fermentable organic matter; VFA, volatile fatty acids.

The analysis of the multi‐kingdom rumen microbiota revealed that inoculation with fresh rumen fluid in early life had long‐lasting effects. As a result, RFF and RFC animals exhibit higher overall microbial diversity than in CTL animals in terms of Shannon's and Simpson's indexes (Figures 2A,B and S1). The inoculation with AUT rumen fluid resulted in intermediate diversity values. The Permanova analysis underscored clear differences in the overall rumen microbial community structure across all treatments, with the exception being RFF and RFC animals. These two groups exhibited a similar microbial community structure that was positively correlated with the rumen concentration of protozoa, methanogens, ammonia and in vitro gas and CH_4_ production (Figure 2C). On the contrary, the overall rumen microbial structures from CTL and AUT animals were associated to forage intake and rumen lactate concentration, respectively.

### 3.4 Rumen Bacteria

The bacterial diversity analysis revealed notable distinctions among the treatment groups (Table 3). The RFF animals displayed the highest rumen bacterial diversity, both in terms of OTUs and Shannon index, followed by RFC animals. In contrast, AUT and CTL animals exhibited the lowest diversity values. These differences in bacterial diversity were reflected in the overall bacterial community structure, which was significantly impacted by the treatments, except for RFF and RFC animals which had a similar bacterial community (Figure 2C). As a result of these overall differences, the abundances of up to 22 bacterial taxa were significantly affected (Table 3). Control animals had the lowest abundance for the phyla Firmicutes and Bacteroidota, resulting in a similar Firmicutes/Bacteroidota ratio across treatments. Inoculation with RFF promoted the highest abundances of Firmicutes, including the taxa Lachnospiraceae and Anaerovibrio (*p* = 0.036). In contrast, RFC and AUT animals had increased abundance of Prevotellaceae (*p* = 0.029), while CTL animals had the highest abundances for Erysipelatoclostridiaceae, Ruminococcaceae, Proteobacteria and Spirochaetota.

**Table 3:** Rumen bacteria diversity and taxa abundances in 7‐month‐old goats at 21 days after an abrupt dietary shift from a forage diet to a high‐concentrate diet. Goats received early‐life daily inoculations during their first 10 weeks of age with autoclaved rumen fluid (AUT), rumen fluid from adult animals fed a forage diet (RFF) or concentrate diet (RFC), or no inoculation (CTL).

	CTL	AUT	RFF	RFC	SED	*p* value
Bacteria diversity						
Richness (OTUs)	208	196	329	272	51.00	0.051
Shannon	2.85^b^	3.11^b^	3.88^a^	3.36^ab^	0.258	0.004
Simpson	0.84^b^	0.88^ab^	0.93^a^	0.89^ab^	0.025	0.012
Bacteria abundances (%)						
Ratio Firmicutes/Bacteroidota	0.61	1.08	1.01	0.39	0.356	0.137
*p_Bacteroidota*	38.7	52.8	45.0	55.3	5.654	0.086
*f_Prevotellaceae*	24.2^b^	43.3^a^	35.3^ab^	46.5^a^	5.792	0.029
*s_bacterium_VCD1001*	0.00^b^	0.01^ab^	0.02^ab^	0.08^a^	0.030	0.034
*p_Firmicutes*	19.7^b^	33.9^ab^	38.8^a^	20.3^b^	6.534	0.044
*g_Family_XIII_AD3011_group*	0.00^b^	0.00^b^	0.01^ab^	0.03^a^	0.012	0.022
*f_Clostridia_vadinBB60_group*	0.10^a^	0.01^b^	0.16^a^	0.02^ab^	0.045	0.046
*f_Erysipelatoclostridiaceae*	2.97	0.01	0.55	0.11	1.021	0.053
*g_UCG‐004*	2.94^a^	0.00^b^	0.52^ab^	0.11^b^	1.010	0.023
*f_Lachnospiraceae*	5.53^b^	7.03^b^	12.59^a^	5.33^b^	2.146	0.036
*g_Acetitomaculum*	0.02	0.11	0.13	0.06	0.035	0.086
*g_Lachnospiraceae_NK3A20_group*	0.24^b^	1.27^ab^	3.61^a^	0.71^ab^	1.244	0.081
*g_Moryella*	0.02^b^	0.12^ab^	0.28^a^	0.23^ab^	0.102	0.009
*g_Oribacterium*	0.01^b^	0.41^a^	0.27^ab^	0.11^ab^	0.130	0.011
*f_Ruminococcaceae*	5.09^a^	0.23^b^	2.12^ab^	0.50^b^	1.406	0.019
*g_Ruminococcus*	5.06^a^	0.18^b^	1.03^b^	0.28^b^	1.351	0.011
*s_Ruminococcus_flavefaciens*	0.16	0.01	0.20	0.04	0.075	0.057
*f_Selenomonadaceae*	2.21^b^	23.5^a^	15.7^ab^	10.8^ab^	5.846	0.009
*g_Anaerovibrio*	0.15^b^	4.77^a^	4.78^a^	1.05^ab^	2.454	0.036
*p_Proteobacteria*	35.8^a^	11.0^b^	12.4^b^	21.8^b^	5.761	0.035
*f_Succinivibrionaceae*	35.2^a^	11.0^b^	11.7^b^	21.4^b^	5.725	0.035
*p_Spirochaetota, g_Treponema*	1.66	0.78	1.86	0.45	0.516	0.094
*s_Treponema_ruminis*	0.87^a^	0.02^b^	0.15^b^	0.06^b^	0.156	0.003

*Note:* Means within a row with different superscript differ (*p* < 0.05). Only phyla (p), families (f), genus (g) and species (s) with an average abundance higher than 0.01% and *p* < 0.1 are shown.

Abbreviation: OUTs, operational taxonomic units.

Correlation analysis (Table S3) identified up to 68 bacterial taxa that correlated with rumen fermentation and feed utilization parameters. Particularly, rumen ammonia concentration showed positive correlations with 19 bacterial taxa, with special interest in *Anaerovora*x and *Lachnospiraceae_NK3A20_group*, but only negative correlations with two species, namely *Treponema ruminis* and *Sphaerochaeta*. Similarly, rumen total VFA concentration had positive correlations with 16 bacterial taxa, including CAG‐352 and Succinivibrionaceae_UCG‐002, while butyrate molar proportion positively correlated with 11 taxa, including *Lachnospiraceae*, with a much lower number of negative correlations (0 and 3, respectively). On the contrary, 12 bacterial taxa showed negative correlations with DMI (including *Olsenella umbonata* and *Erysipelatoclostridiaceae*) and rumen propionate molar proportion (including *Succiniclasticum* and *Lachnospiraceae_NK3A20_group*). Other analysed parameters displayed a more limited number of correlations with bacterial taxa.

### 3.5 Rumen Methanogens

Inoculating fresh rumen fluid during early life had enduring effects on the rumen methanogens community structure which resulted to be positively correlated with the presence of rumen protozoa, (Figure 2E). This also resulted in higher methanogens richness (*p *= 0.003) and Shannon index (*p *= 0.078) compared to CTL animals (Table 4). The rumen methanogens community was mostly dominated by *Methanomassiliicoccaceae* (50% of the community) and *Methanobacteriaceae* (49%). Interestingly, the abundance of most methanogenic taxa remained unaffected by the treatments. Only two species, *Candidatus_Methanomethylophilus_alvus* and *Group11_spISO4‐G11*, were more abundant in CTL animals than in the other treatment groups. Correlation analysis (Table S3) showed that the concentration and richness of methanogens, as well as the most abundant species, *Methanobrevibacter gottschalkii*, showed negative correlations with rumen propionate molar proportion. This highlights the complex interplay between rumen methanogens and rumen fermentation parameters, particularly with respect to propionate production.

**Table 4:** Rumen methanogens diversity and taxa abundances in 7‐month‐old goats at 21 days after an abrupt dietary shift from a forage diet to a high‐concentrate diet. Goats received early‐life daily inoculations during their first 10 weeks of age with autoclaved rumen fluid (AUT), rumen fluid from adult animals fed a forage diet (RFF) or concentrate diet (RFC), or no inoculation (CTL).

	CTL	AUT	RFF	RFC	SED	*p* value
Methanogens diversity						
Richness (OTUs)	8.80^b^	9.17^b^	16.4^a^	10.8^ab^	1.816	0.003
Shannon	1.08	1.21	1.67	1.45	0.222	0.078
Simpson	0.53	0.58	0.70	0.68	0.095	0.311
Methanogens abundances (%)						
*f_Methanobacteriaceae; g_Methanobrevibacter*	38.2	57.6	56.8	47.6	8.786	0.750
*s_Methanobrevibacter_boviskoreani_clade*	14.1	21.2	24.6	10.5	6.653	0.536
*s_Methanobrevibacter_gottschalkii_clade*	19.5	28.7	27.1	33.6	7.398	0.946
*s_Methanobrevibacter_oralis*	0.00	0.00	0.02	0.08	0.021	0.370
*s_Methanobrevibacter_ruminantium_clade*	4.48	7.64	2.15	1.89	2.083	0.656
*s_Methanobrevibacter_smithii*	0.00	0.00	0.00	0.01	0.004	0.522
*f_Methanomicrobiaceae; g_Methanomicrobium*	0.00	0.03	0.00	0.00	0.011	0.438
*f_Methanomassiliicoccaceae*	61.8	42.4	41.8	52.3	8.780	0.732
*s_Candidatus_Methanomethylophilus_alvus*	30.0^a^	0.00^b^	0.64^b^	0.00^b^	5.000	0.012
*s_Group8_spWGK1*	0.04	0.26	8.35	0.34	1.620	0.173
*s_Group9_sp*	0.00	0.15	0.08	0.00	0.054	0.555
*s_Group9_spCH1270*	0.00	0.00	0.02	0.00	0.004	0.343
*s_Group9_spISO4‐G1*	10.6	14.8	10.0	10.0	5.291	0.748
*s_Group10_sp*	6.92	1.69	1.57	3.19	1.872	0.725
*s_Group11_spISO4‐G11*	9.00^a^	0.00^b^	1.87^b^	0.29^b^	1.648	0.036
*s_Group12_spISO4‐H5*	4.36	22.17	5.56	38.0	7.291	0.129

*Note:* Means within a row with different superscript differ (*p* < 0.05).

Abbreviation: OUTs, operational taxonomic units.

### 3.6 Rumen Protozoa and Anaerobic Fungi

Protozoal sequencing revealed lower protozoal diversity indexes for AUT compared to RFF and RFC animals (Table 5). Additionally, PCoA and PERMANOVA analyses identified differences in the protozoal community structure between AUT and RFF or RFC animals (*p *< 0.001, Figure 1F). The protozoal community structure in RFF and RFC animals appeared similar and was positively correlated with the diversity of rumen bacteria, methanogens, and protozoa. It was also positively associated with the total VFA concentration and butyrate molar proportion. In contrast, the protozoal community in AUT animals was dominated (97.6%) by entodiniomorphids (family Ophyoscolecidae), while RFF had an increased concentration of holotrichs (33.2%), such as Isotricha, and RFC displayed an intermediate value. In the correlation analysis (Table S3), it was evident that rumen ammonia concentration had strong positive correlations with the rumen protozoal concentration, richness, and the abundance of the most relevant entodiniomorphid taxa, including *Entodinium, Ophryoscolex* and *Polyplastron*. Furthermore, protozoal concentration and *Ophryoscolex* abundance exhibited positive correlations with butyrate and negative correlations with propionate molar proportions. Both Isotricha species (*Isotricha prostoma* and *Isotricha intestinalis*) were positively correlated with the rumen total VFA concentration.

**Table 5:** Rumen protozoa and anaerobic fungi diversity and taxa abundance in 7‐month‐old goats at 21 days after an abrupt dietary shift from a forage diet to a high‐concentrate diet. Goats received early‐life daily inoculations during their first 10 weeks of age with autoclaved rumen fluid (AUT), rumen fluid from adult animals fed a forage diet (RFF) or concentrate diet (RFC), or no inoculation (CTL).

	CTL	AUT	RFF	RFC	SED	*p* value
Protozoa diversity						
Richness (OTUs)	ND	15.8^b^	23.5^a^	22.0^a^	2.316	0.007
Shannon	ND	1.41^b^	1.84^a^	1.82^a^	0.183	0.034
Simpson	ND	0.65	0.74	0.76	0.065	0.134
Protozoal abundances (%)						
*f_Ophyoscolecidae*	ND	97.6^a^	65.9^b^	87.4^ab^	5.021	< 0.001
*g_Entodinium*	ND	80.7^a^	37.7^b^	70.2^a^	6.668	0.002
*g_Diplodinium*	ND	0.01^a^	0.00^b^	0.00^b^	0.001	0.038
*g_Polyplastron*	ND	16.9	28.0	17.1	5.226	0.493
*g_Enoploplastron*	ND	0.00	0.10	0.07	0.042	0.378
*g_Ophryoscolex*	ND	0.00^b^	0.12^a^	0.03^ab^	0.029	0.099
*f_Isotrichidae*	ND	0.03^c^	33.2^a^	10.7^b^	5.071	< 0.001
*g_Dasytricha*	ND	0.01^b^	2.63^a^	2.41^a^	0.978	0.017
*g_Isotricha*	ND	0.01^c^	30.6^a^	8.27^b^	4.679	< 0.001
*s_Isotricha_prostoma*	ND	0.01^c^	23.0^a^	7.48^b^	3.630	< 0.001
*s_Isotricha_intestinalis*	ND	0.00^c^	5.19^a^	0.37^b^	1.113	< 0.001
Anaerobic fungy diversity						
Richness (OTUs)	30.4	31.7	31.3	30.8	5.250	0.992
Shannon	1.77	2.10	1.73	1.84	0.259	0.471
Simpson	0.70	0.81	0.65	0.72	0.079	0.244
Fungal abundances (%)						
*f_Neocallimastigaceae spp*.	87.5	71.2	67.5	71.3	5.196	0.432
*g_Anaeromyces*	0.36^b^	12.6^a^	0.00^b^	0.79^b^	2.351	0.047
*g_Caecomyces*	0.04	2.67	2.39	9.70	2.438	0.139
*g_Neocallimastix*	12.1	13.5	30.1	18.2	4.584	0.421

*Note:* Means within a row with different superscript differ (*p* < 0.05).

Abbreviations: ND, no detected; OUTs, operational taxonomic units.

The experimental treatments had a relatively minor impact on the anaerobic fungal community structure. Similar fungal diversity indexes and taxa abundances were observed across treatments, but AUT animals had the highest abundance for *Anaeromyces* (Table 5). The PCoA analysis showed minor differences in the rumen fungal community structure across treatments being the community of the inoculated animals positively correlated with total VFA concentration and negatively with rumen pH (Figure 2G). The rumen anaerobic fungal concentration showed a positive correlation with acetate production and a negative correlation with propionate production, reflecting the role of anaerobic fungi in the fermentation process (Table S3). Furthermore, fungal richness and the abundance of *Anaeromyces* were positively correlated with NDF digestibility, suggesting a potential influence of these fungi on fibre degradation and utilization in the rumen.

### 3.7 Hindgut Microbiota and Fermentation

**Figure 3:**
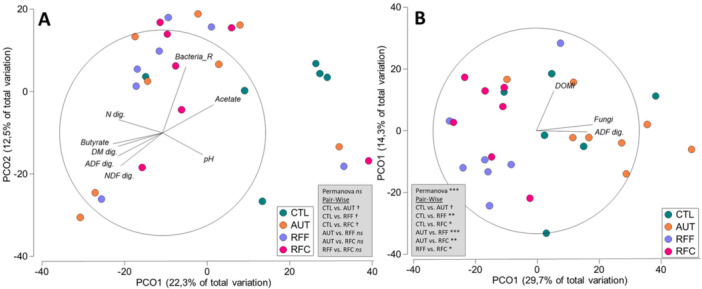
Principal coordinates analysis illustrating relationships (*ρ* > 0.4) between the structure of the caecal bacterial (A) and methanogens (B) community and caecal fermentation and digestibility data in in 7‐month‐old goats at 21 days after an abrupt dietary shift from a forage diet to a high‐concentrate diet. Goats received early‐life daily inoculations during their first 10 weeks of age with autoclaved rumen fluid (AUT), rumen fluid from adult animals fed a forage diet (RFF) or concentrate diet (RFC), or no inoculation (CTL). PERMANOVA values are provided based on the Bray‐Curtis dissimilarity. ADF, acid detergent fibre; DOM, digestible organic matter; NDF, neutral detergent fibre.

In the context of hindgut microbial fermentation, the experimental treatments did not have a significant impact on various parameters, including caecal pH, VFA concentration, bacterial concentration and bacterial diversity (Table 6). However, there were subtle differences in the hindgut bacterial community composition, particularly in the inoculated animals (AUR, RFF and RFC), when compared to CTL animals (Figure 3A). These differences in bacterial community structure were associated with fibre digestion and butyrate production. Despite the Firmicutes/Bacteroidota ratio remaining constant, there were tendencies for up to 13 bacterial taxa to differ across treatments (Table 6). Most of them were minority taxa, but they showed lower abundance in CTL animals compared to RFF animals, including species like *Faecalibacterium*, *Ruminococcaceae* and *Oscillospira*. In contrast, CTL animals had higher abundances for *Lachnospiraceae_UCG‐010* and *Treponema_berlinense*.

**Table 6:** Hindgut fermentation and microbial diversity and taxa abundances in 7‐month‐old goats at 21 days after an abrupt dietary shift from a forage diet to a high‐concentrate diet. Goats received early‐life daily inoculations during their first 10 weeks of age with autoclaved rumen fluid (AUT), rumen fluid from adult animals fed a forage diet (RFF) or concentrate diet (RFC), or no inoculation (CTL).

	CON	PRE	RLF	RLC	SED	*p* value
Caecal pH	6.16	6.22	6.16	6.18	0.133	0.968
Total VFA (mmol/L)	102	98.9	101	101	9.195	0.997
Molar proportions (%)						
Acetate	72.7	68.4	70.9	69.2	2.244	0.241
Propionate	13.8	15.2	13.9	13.4	0.854	0.169
Butyrate	10.2	13.4	12.6	15.0	2.305	0.292
Valerate	1.38	1.33	1.20	1.06	0.160	0.201
BCVFA	1.93	1.68	1.31	1.40	0.322	0.226
Bacteria						
Concentration^a^ (log10 copy/mg DM)	11.72	11.66	11.64	11.65	0.125	0.805
Richness (OTUs)	232	223	239	237	38.00	0.956
Shannon	3.30	3.03	3.47	3.28	0.361	0.627
Simpson	0.85	0.81	0.92	0.89	0.071	0.403
Bacterial abundances (%)						
Firmicutes/bacteroidota ratio	0.73	0.59	0.90	0.73	0.148	0.805
*p_Bacteroidota; s_Bacteroides_sartorii*	0.03^b^	0.35^a^	0.00^b^	0.01^b^	0.124	0.004
*p_Bacteroidota; s_Bacteroides_vulgatus*	0.14	0.08	0.08	0.03	0.065	0.091
*p_Bacteroidota; f_Barnesiellaceae*	0.12^b^	0.40^a^	0.54^a^	0.14^b^	0.186	0.041
*p_Bacteroidota; s_Alistipes_finegoldii*	0.00^b^	0.02^ab^	0.15^a^	0.03^ab^	0.055	0.045
*p_Bacteroidota; g_Parabacteroides*	0.25	0.23	0.46	0.03	0.193	0.053
*p_Firmicutes; g_Breznakia*	0.01	0.02	0.05	0.09	0.025	0.065
*p_Firmicutes; g_Acetitomaculum*	0.02^b^	0.77^a^	0.31^ab^	0.12^ab^	0.279	0.005
*p_Firmicutes; g_Lachnospiraceae_UCG‐010*	0.10	0.02	0.03	0.00	0.024	0.059
*p_Firmicutes; g_Oscillospira*	0.00	0.03	0.07	0.02	0.020	0.096
*p_Firmicutes; g_Faecalibacterium*	0.93	4.25	4.10	7.23	1.935	0.083
*p_Firmicutes; f_Ruminococcaceae*	2.47	5.79	6.91	9.38	2.053	0.084
*p_Proteobacteria; g_Escherichia‐Shigella*	0.00^b^	0.11^a^	0.08^a^	0.00^b^	0.048	0.009
*p_Spirochaetota; s_Treponema_berlinense*	0.79^a^	0.02^b^	0.25^ab^	0.00^b^	0.266	0.025
Methanogens						
Concentration^a^ (log10 copy/mg DM)	7.78^b^	8.42^a^	8.39^a^	8.35^a^	0.164	0.013
Richness (OTUs)	13.2^bc^	9.88^c^	17.9^ab^	20.6^a^	2.699	0.004
Shannon's index	1.42^bc^	1.08^c^	1.73^ab^	1.88^a^	0.206	0.004
Simpson's index	0.66^ab^	0.50^b^	0.75^a^	0.78^a^	0.078	0.007
Methanogens abundances (%)						
s_Methanobrevibacter_oralis	0.02	0.06	0.25	0.14	0.038	0.057
g_Methanomassiliicoccaceae; g_Gr.9	0.62^a^	0.00^b^	0.03^b^	10.8^b^	1.851	0.026
g_Methanomassiliicoccaceae; s_Gr.9_sp	0.00^b^	0.00^b^	0.03^b^	2.12^a^	0.393	0.002
g_Methanomassiliicoccaceae; s_Gr.9_spISO4‐G1	0.00^b^	0.00^b^	0.01^b^	8.64^a^	1.716	0.002
g_Methanomassiliicoccaceae; s_Gr.12_spISO4‐H5	0.00^b^	3.24^ab^	15.9^a^	9.68^a^	3.158	0.019
Anaerobic fungi (log10 copy/mg DM)	5.09	4.95	4.92	4.88	0.097	0.099

Abbreviations: BCVFA, branched chain VFA; OUTs, operational taxonomic units; VFA, volatile fatty acids.

*Note:* Means within a row with different superscript differ (*p* < 0.05). Only phyla (p), families (f), genus (g) and species (s) with an average abundance higher than 0.01% and *p* < 0.1 are shown.

^a^ Caecal concentration in log10 (copies/mg DM) based on qPCR data.

The hindgut methanogenic community was notably influenced by the experimental treatments (Table 6). The PCoA also confirmed differences in the methanogens community structure across all treatments (Figure 3B). As a result, the inoculated animals (RFF and RFC) had a higher hindgut concentration of methanogens (*p* = 0.013), but it was accompanied by a lower methanogens diversity (*p* = 0.004). Moreover, RFF and RFC animals showed increased abundances of *Methanobrevibacter_oralis* (*p* = 0.057) and 4 Methanomassiliicoccaceae species including *Group9* (*p* = 0.026) and *Group12_spISO4‐H5* (*p* = 0.019). The anaerobic fungal concentration in the hindgut tended to be higher for CTL than for the animals subjected to other treatments (*p* = 0.099).

### 3.8 Gut Anatomical Development and Gene Expression

The gut's anatomical development remained largely unaffected by the experimental treatments, and the sizes of various organs, including the total gastrointestinal tract (GIT), rumen, small intestine and large intestine, were similar across treatments when expressed as a % of BW (Table 7). However, a more detailed microscopic examination of the rumen papillae revealed subtle differences. Inoculated animals tended to have slightly shorter and wider rumen papillae compared to CTL animals (*p* = 0.091), resulting in a lower length‐to‐width ratio (*p* = 0.002).

**Table 7:** Gut anatomical development and gene expression in 7‐month‐old goats at 21 days after an abrupt dietary shift from a forage diet to a high‐concentrate diet. Goats received early‐life daily inoculations during their first 10 weeks of age with autoclaved rumen fluid (AUT), rumen fluid from adult animals fed a forage diet (RFF) or concentrate diet (RFC), or no inoculation (CTL).

	CTL	AUT	RFF	RFC	SED	*p* value
Total full GIT, % BW	22.5	21.4	24.1	22.4	1.225	0.439
Full rumen, % BW	11.2	10.4	12.0	11.1	0.794	0.184
Empty rumen, % BW	2.15	2.20	2.21	2.27	0.122	0.794
Full omasum, % BW	0.47	0.42	0.42	0.45	0.064	0.708
Full abomasum, %BW	1.77	1.42	1.79	1.74	0.168	0.125
Full small intestine, % BW	5.60	5.65	6.20	5.64	0.366	0.332
Full large intestine, % BW	3.50	3.52	3.69	3.46	0.258	0.703
Rumen papillae						
Length (µm)	612	579	488	670	51.80	0.121
Width (µm)	90.9	128	142	119	12.30	0.091
Ratio length/width	7.09^a^	4.62^bc^	3.69^c^	5.82^ab^	0.534	0.002
Surface (mm^2^)	0.19	0.26	0.24	0.27	0.034	0.549
Rumen epithelium						
IGFR1	1.00^b^	1.06^ab^	1.34^a^	0.95^b^	0.143	0.050
Cyclin A	1.00^c^	1.56^ab^	2.00^a^	1.32^bc^	0.270	0.009
Cyclin D1	1.00	1.75	1.61	1.54	0.428	0.349
CDK2	1.00	0.85	1.30	1.63	0.534	0.495
BCL2	1.00	0.57	0.85	0.81	0.175	0.126
BAX	1.00	1.11	1.25	1.13	0.200	0.700
Caspase‐3	1.00	1.44	1.72	1.59	0.368	0.271
Bcl‐2/Bax	1.00	0.56	0.67	0.71	0.196	0.169
MCT1	1.00^b^	5.25^a^	5.98^a^	4.41^a^	1.231	0.002
PAT1	1.00	1.33	1.58	1.54	0.396	0.476
BDH1	1.00	1.14	1.19	1.01	0.241	0.822
HMGCS1	1.00	1.24	1.56	1.55	0.392	0.452
HMGCL	1.00^b^	2.95^a^	2.64^a^	2.34^a^	0.574	0.012
Caecal epithelium						
IGFR1	1.00	0.77	0.17	0.66	0.468	0.390
CICLYN A	1.00	0.81	0.43	0.43	0.311	0.190
MCT1	1.00^a^	0.01^b^	0.00^b^	0.01^b^	0.331	0.014
PAT1	1.00^a^	0.02^b^	0.00^b^	0.00^b^	0.302	0.013
BDH1	1.00^a^	0.01^ab^	0.00^c^	0.00^c^	0.003	< 0.001
HGMCS1	1.00	0.04	0.01	1.10	0.654	0.245
HGMCL	1.00	0.72	0.01	0.24	0.536	0.361

*Note:* Gene expression was calculated based on the $\Delta \Delta C_{t}$ method (Kenneth and Schmittgen 2001) using the β‐actine as housekeeping gene. Means within a raow with different superscript differ (*p* < 0.05).

Abbreviations: BW, body weight; GIT, gastrointestinal tract.

Gene expression analysis of the rumen epithelium provided insights into the functional implications of these anatomical differences. Inoculated animals (AUT, RFF and RFC) exhibited higher expression levels of genes related to rumen energy uptake, including *Cycling A* (*p* = 0.009), *MCT1* (*p* = 0.002) and *HMGCL* (*p* = 0.012). These genes are involved in metabolic pathways associated with cell proliferation, volatile fatty acid (VFA) absorption, and VFA metabolism, respectively. Additionally, RFF animals tended to have increased expression of the *IGFR1* gene (*p* = 0.055), which is associated with rumen epithelial growth. In contrast, gene expression analysis in the caecal epithelium revealed a different scenario. Inoculated animals exhibited lower expression of genes related to VFA absorption, such as *MCT1* (*p* = 0.014) and *PAT1* (*p* = 0.013), as well as genes related to VFA metabolism, including *BDH1* (*p* < 0.001). These findings suggest that inoculated animals had a lower energy uptake by the caecal enterocytes compared to the CTL animals.

## 4 Discussion

### 4.1 Effects of Different Microbial Inocula

Adult ruminants exhibit a high degree of host specificity, which represents a challenge when attempting to bring out permanent modifications in their rumen microbiota (Weimer 2015). In contrast, it has been proposed that nutritional interventions during the early stages of ruminant's life offer a unique opportunity to influence the rumen microbial colonization process. Such interventions can yield both short‐term and potentially long‐term effects on the structure of the rumen microbial community and, consequently, on animal productivity (Yáñez‐Ruiz et al. 2015). In a companion publication from the present experiment, we described the advantages of early‐life inoculation of young ruminants with fresh rumen fluid, illustrating how it could accelerate the development of the rumen microbial ecosystem and optimize the weaning process at 7 weeks of age (Palma‐Hidalgo et al. 2021). This current publication delves further into the persistency of these effects. Even after a substantial time elapsed (31 weeks), the animals that received the rumen fluid (RFF and RFC) still displayed a significantly higher richness in their rumen multi‐kingdom microbial community compared to the CTL group (+209 and +114 OTUs, respectively). This higher overall richness was a consequence of greater bacterial, methanogens, and protozoal diversity, while the CTL animals remained protozoa‐free. The low rumen microbial diversity and absence of rumen protozoa observed in the CTL animals are commonly found in artificially reared ruminants (Belanche, Yáñez‐Ruiz et al. 2019). This absence of protozoa is attributed to their high sensitivity to oxygen, which implies that a direct contact with adult ruminants is needed for an effective protozoal transmission (Palma‐Hidalgo, Yáñez‐Ruiz et al. 2021). In contrast, the animals from the AUT group, when compared to those inoculated with fresh rumen fluid, retained lower bacterial, methanogens and protozoal diversity, and their rumen microbiota was primarily dominated by only two genera (*Entodinium* and *Polyplastron*). This observation raises the possibility of an incomplete rumen protozoal colonization, possibly due to an accidental cross‐faunation event during animal management. Notably, the AUT animals appeared to undergo a more favourable diet adaptation process compared to the CTL lambs and resulting in lower rates of diarrhoea and higher BW gain. These benefits could be attributed to the presence of rumen protozoa, even in low diversity, and the potential positive effects derived from the inoculation with rumen fermentation products like butyrate (O'Hara et al. 2018), micro‐nutrients and microbial extracts which can favour the rumen papillae development (Muscato et al. 2002; Zhong et al. 2014). Interestingly, this study showed a lower rumen multi‐kingdom diversity when animals were adapted to a high‐concentrate diets (average 333 OTUs) than a companion paper in which these animals were fed a full forage diet (average 445 OTUs, Belanche, Palma‐Hidalgo et al. 2023), indicating that forage degradation in the rumen requires a more complex and diverse rumen microbiota as previously described in sheep (Belanche, Kingston‐Smith et al. 2019). The fact that all animals had a similar DMI and feed digestibility values across treatments, while CTL animals had a had a loss in BW, inoculated animals had a BW gain during the 21 days after the dietary shift seems to indicate a more successful adaptation of these latter animals. This could be partially explained by a potential decrease in the rumen content of the CTL animals, as it has been suggested that defaunated animals tend to have a higher rumen volume particularly when fed forage diet as a result of lower fibre digestibility and higher rumen retention time (Newbold et al. 1995). This hypothesis seems to be supported by the observed lower forage consumption in the CTL animals during the 4 days after the dietary shift.

In relation to the type of inoculum used, previous studies provide insights into the potential benefits of using rumen fluids with different characteristics. Muscato et al. (2002) reported that dairy calves inoculated with either fresh or autoclaved rumen fluids exhibited higher BW gain and experienced fewer faecal scours during the post‐weaning period. Huuki et al. (2022) reported that calves inoculated with fresh rumen fluid from adult cows had higher DMI in early life as a result of higher rumen microbial development as well as a more homogeneous milk production later in life than their non‐inoculated monozygotic twins. Similarly, Zhong et al. (2014) observed increased feed digestibility and BW gain in lambs inoculated with either fresh or lyophilized rumen fluid. Furthermore, scientific evidence suggests that the inoculation of young lambs with fresh rumen fluid from adult ewes adapted to various diets, such as coconut oil or protected fat, can effectively modulate the rumen microbiota (De Barbieri, Hegarty, Silveira et al. 2015) and improve rumen fermentation patterns during the post‐weaning period (De Barbieri, Hegarty, Silveira and Oddy 2015). Therefore, we hypothesized that inoculation with a rumen inoculum adapted to a high‐concentrate diet (RFC) could be beneficial during the adaptation process and subsequent utilization of such a diet in adult life.

Our study suggests that the initial differences observed in rumen microbial colonization between RFF and RFC animals (Palma‐Hidalgo, Jiménez et al. 2021) tended to diminish later in life, resulting in a similar overall rumen microbial community structure. This microbial convergence underscores the remarkable adaptability of the rumen microbiota to dietary changes (Weimer 2015). However, RFC animals exhibited lower bacterial and methanogens richness compared to RFF animals, which was consistent with the characteristics of the RFC inoculum. Furthermore, differences were also observed in the abundance of specific rumen (e.g., Lachnospiraceae) and caecal bacterial taxa (e.g., Parabacteroides, Barnesiellaceae, and *Escherichia‐Shigella*). These microbial distinctions could explain why RFC animals demonstrated a slightly better adaptation to the high‐concentrate diet, as evidenced by a lower incidence and prevalence of diarrhoea and higher BW gain. These findings suggest that partial rumen microbial programming through early‐life nutritional interventions to enhance animal resilience should not be dismissed (Yáñez‐Ruiz et al. 2015).

### 4.2 Adaptation Process to a High‐Concentrate Diet

A sudden decline in rumen pH can trigger a rumen acidosis which represents a significant health issue in dairy farming, especially when transitioning from a forage‐based diet to a high‐concentrate diet. This abrupt shift often leads to reduced feed intake, digestive issues (i.e., rumen acidosis and diarrhoea), health problems, decreased productivity, and increased management and veterinary costs (Owens et al. 1998). To mitigate this issue, various strategies have been proposed, including the use of pH buffers (Keunen et al. 2003), phytochemicals (Rivera‐Chacon et al. 2022), or the implementation of a gradual diet transition (Owens et al. 1998). Furthermore, it is well‐established that the rumen microbiota plays a pivotal role in the adaptation process during such dietary shifts (Ramos et al. 2021). Brown et al. (2006) noted substantial inter‐animal variability in the ability to cope with high‐concentrate diets. However, they also found that a portion of this variation, favouring successful adaptation, could be attributed to the maintenance of rumen protozoa and lactate‐utilizing bacteria. As a result, we hypothesized that optimizing the adaptation process to high‐concentrate diets could be achieved through a deliberate modulation of the rumen microbiota.

To investigate this question, our experimental animals underwent a challenge involving a sudden transition to a high‐concentrate diet, aiming to assess the potential impact of inoculation on the dietary adaptation process. Throughout this adaptation period, it was observed a gradual increase in total DMI (+41%), rumen VFA (+16%) and lactate concentration (+33%), and a decrease in rumen pH by −0.21 units from Day 4 to 21. These rumen pH values were within the range (5.23–6.69) previously reported for goats fed concentrate rich diets (Belanche, Arturo‐Schaan et al. 2023). Moreover, most carbohydrates from the concentrate diet seemed to be fermented in the rumen as lower blood glucose values were observed in this study than reported when goats were fed forage (average 1.92 vs. 3.0 mmol/L) (Belanche, Palma‐Hidalgo et al. 2023). Notably, the inoculation with fresh rumen fluid appeared to expedite this adaptation process, maintaining rumen pH at higher values (an average of 6.44) compared to the control (CTL) animals (average of 5.94) after the dietary shift. Additionally, the inoculation promoted a gradual increase in protozoal concentration (+8%). Rumen protozoa, primarily holotrichs, have the capacity to engulf and accumulate starch grains and soluble carbohydrates (Williams and Coleman 1992), effectively preventing alternative bacterial fermentation that could lead to decreased pH and the onset of lactic acid acidosis (Mackie et al. 1978). It is worth noting that when rumen pH remains below 6, it has been suggested that ruminants can experience sub‐acute rumen acidosis, which often results in irregular feed consumption, weight loss, and reduced animal performance (Owens et al. 1998). It has been described that sub‐acute rumen acidosis increases plasma acute phase proteins and cortisol in goats (Jia et al. 2014), an aspect that was not observed in our study. Fulton et al. (1979), in a study involving the infusion of buffers into the rumen, identified a correlation between low rumen pH and the cessation of voluntary feed intake in beef cattle, subsequently leading to lower performance. Our study partially agreed with these findings, as animals in the inoculated groups (AUT, RFF and RFC) tended to exhibit a positive BW gain during the diet adaptation process, whereas the CTL animals experienced a BW loss and a lower rumen pH during such period. Furthermore, up to 83% of the CTL animals exhibited signs of diarrhoea, with an average duration of 12 days, whereas it was negligible for the RFF and RFC groups. A recent study (Zhang et al. 2022) revealed that those dairy cows which were sensitive to rumen acidosis had higher rumen propionate proportions than their tolerant counterparts, differences which were also observed between our CTL and inoculated animals (+16%). These findings suggest that although DMI was similar across treatments, promoting a diverse rumen microbiota, including the presence of protozoa, favoured the adaptation process to high‐concentrate diets and resulted in a reduced risk of rumen acidosis.

### 4.3 Rumen Microbial Fermentation and Feed Utilization

Besides the positive effects observed during the diet adaptation process, this study identified that promoting greater overall rumen microbial diversity can have various long‐term implications on rumen microbial fermentation and feed utilization, with mostly positive and some negative aspects. A comprehensive meta‐analysis involving 48 studies, comparing faunated and defaunated animals (Newbold et al. 2015), illustrated that the presence of rumen protozoa induces changes at multiple levels: (1) at the microbiology level, rumen protozoa promote higher concentrations of bacteria and anaerobic fungi, greater diversity in methanogens, and increased abundances of fibrolytic bacteria, such as *Ruminococcus* and *Fibrobacter*; (2) in terms of energy metabolism, the presence of protozoa leads to increased rumen VFA (mostly butyrate) concentration, and enhance OM degradation and fibre digestion leading to higher methane emissions; and (3) in terms of N metabolism, protozoa promote protein breakdown, elevate rumen ammonia concentration, urinary N excretion and reduce microbial protein flow to the small intestine. As a result of this, presence of protozoa often has adverse effect on productivity, resulting in lower BW and N retention by the host.

Our findings align with most of these observations, emphasizing that the presence of rumen protozoa in the inoculated animals played a pivotal role. Consequently, positive correlations were identified between rumen VFA and holotrich protozoa, between rumen butyrate and protozoal concentration, and between rumen ammonia and various entodinomorphids. Previous research has indicated that concentrate supplementation up to 75% encourages an increase in rumen protozoal concentration, while further increments tend to decrease protozoal numbers, with *Entodinium* being the most resilient protozoa in low rumen pH conditions (Franzolin and Dehority 1996). Moreover, nearly complete rumen defaunation has been reported in some animals fed full‐concentrate diets (Hook et al. 2011). However, the substantial inter‐animal variability in this regard suggests that various factors, such as the rate of feed consumption, passage rate, or salivary production, can also influence the host's ability to cope with high‐concentrate diets (Franzolin and Dehority 1996).

Our study observed that, following the diet adaptation process, the presence of protozoa and the higher overall rumen diversity in RFF and RFC animals had several positive effects on rumen feed fermentation. This resulted in increased VFA production by 16% and a substantial rise in butyrate production by 60%. Notably, in vitro methane (CH_4_) production also increased by 34%, confirming a previously described linear relationship between CH_4_ emissions and protozoal concentration (Guyader et al. 2014). However, it is interesting to note that our study did not detect any significant differences in terms of feed digestibility, N balance, or microbial protein synthesis across the experimental treatments. Ivan et al. (2000) demonstrated that the progressive inoculation of fauna‐free ewes with major individual species of ciliate protozoa reduced the efficiency of N utilization, particularly when the ruminant was fed a forage‐diet, leading to lower microbial protein flow to the intestine (−23%). This was primarily attributed to the engulfment of ruminant bacteria by different species of rumen protozoa. Holotrich protozoa, such as *Isotricha* and *Dasytricha*, had minimal effects on N metabolism in the rumen, whereas the large entodiniomorphids (including *Diplodinium, Polyplastron, Epidinium, Ophryoscolex, Eudiplodinium*) and *Entodinium* have a negative impact on the N utilization by the host (Ivan et al. 2000; Belanche et al. 2012). Additionally, higher protein breakdown was detected in the RFF and RFC animals, which had ammonia concentrations in the rumen that were 2.5 times higher than those in the CTL animals. However, given the high protein content in the diet (averaging 17.2%), the ammonia levels always remained above the theoretical threshold (50 mg/L) that could limit microbial protein synthesis in the rumen (Calsamiglia et al. 2010). This indicates that the availability of rumen‐degradable N was not a limiting factor in our experiment. As a result, up to 19 bacterial taxa displayed a positive correlation with rumen ammonia concentration, including the Prevotellaceae family, which comprises the majority of bacteria with dipeptidyl peptidase activity, considered a bottleneck in the rumen proteolysis process and further N incorporation into microbial protein (Wallace et al. 1997). Similarly, the lack of differences in feed digestibility might be attributed to the presence of a high proportion of highly fermentable carbohydrates in the diet and a low forage‐to‐concentrate ratio (22:78). Moreover, the lack of differences in the hindgut fermentation suggests that most of the nutrients were fermented in the rumen. These findings collectively suggest that promoting a high rumen microbial diversity does not have positive effects on the rumen microbial fermentation and the feed utilization when animals are fed highly fermentable diets, and there is no shortage of degradable N available for the rumen microbes.

The positive correlation observed in up to 16 bacterial taxa with rumen VFA concentrations suggests a significant role of rumen bacteria in carbohydrate fermentation. Notably, in terms of easily fermentable carbohydrates, the absence of protozoa in CTL animals was largely compensated by an increased abundance of specific amylolytic and cellulolytic bacteria, such as the families Succinivibrionaceae (2.4‐fold higher), Erysipelatoclostridiaceae (13.4‐fold higher) and Ruminococcaceae (5.4‐fold higher). Succinivibrionaceae genera, including *Succinivibrio dextrinosolvens* and *Succinomonas amylolytica*, can tolerate low rumen pH and ferment sugars into acetate, lactate, formate, and succinate (Hespell 1992). Members of the Ruminococcaceae family, including *Ruminococcus albus* and *Ruminococcus flavefaciens*, are able to utilize a wider range of substrates including cellulose, hemicellulose and pectin (*R. albus* also starch and glucose) and producing acetate and lactate as main fermentation products (Hespell 1992). In contrast, inoculated animals (AUT, RFF and RFC) exhibited an increased abundance of Selenomonadaceae (16.7‐fold higher), which includes saccharolytic bacteria like *Selenomonas ruminantium*, capable of fermenting starch, lactate, and soluble sugars into VFA. Recent findings (Xue et al. 2022) employing integrated meta‐omics approaches revealed that in the rumen of highly efficient cows, *Selenomonas* and members of the Succinivibrionaceae family positively interacted with each other, acting as keystone members due to their essential ecological functions on the carbohydrate degradation process. Moreover, a detailed rumen microbiome and metabolome study demonstrated that dairy cows susceptible to rumen acidosis (as our CTL animals) had different rumen microbial communities including more starch‐degrading bacteria and fewer fibre‐degrading bacteria resulting on a higher propionate production (Zhang et al. 2022) as noted in our study. Thus, our study builds upon these observations, indicating that the abundance of these bacterial taxa in the rumen can be modulated through early‐life nutritional interventions.

Another study (Shabat et al. 2016), involving cows fed a high‐concentrate diet (30:70 forage to concentrate ratio), reported that feed‐efficient cows had lower rumen bacterial diversity, lower CH_4_ emissions, higher propionate molar proportion (as seen in our CTL animals), but also had higher levels of butyrate, *Megasphaera* and *Lachnospiraceae* in the rumen, which aligns with our RFF and RFC animals. This discrepancy highlights that feed efficiency is a complex trait influenced by factors like diet type, feeding patterns or animal type, among others. Thus, any associations between productive data and specific bacterial taxa represents an oversimplification of the microbial ecology, where other microbial groups (methanogens, protozoa and anaerobic fungi) can also play crucial roles (Newbold et al. 2015). In this sense, the present study represents one of the few that have employed a multi‐kingdom meta‐taxonomic approach to achieve a holistic analysis of the rumen microbiota. As previously mentioned, the overall structure of the entire rumen microbiota was significantly influenced by the presence of rumen protozoa, but each microbial community had its own unique characteristics. For instance, the observed increase in methanogens diversity in RFF and RFC animals could be attributed to the estimation that between 9% and 25% of rumen methanogens are associated with protozoa (Newbold et al. 1995). The positive correlation between the methanogens diversity and butyrate, the main fermentation product derived from protozoal activity, suggests an active role of these two microbial communities in the inter‐species hydrogen transfer during rumen methanogenesis (Morgavi et al. 2010). Similarly, anaerobic fungi diversity and the abundance of *Anaeromyces* showed a positive correlation with NDF digestibility, implying an active role of fungi in fibre degradation (Edwards et al. 2017). Despite these microbial findings, the methanogens' ability to colonize the rumen shortly after birth (Abecia et al. 2013), along with the anaerobic fungi's capacity to form resistant spores that remain viable in the environment (McGranaghan et al. 1999) may explain why early‐life inoculation with rumen fluid had a relatively minor long‐term impact on the overall structure of these two microbial communities.

## 5 Gene Expression, Hindgut Microbial Fermentation and Health

The rumen anatomical development is a process that occur following three phases: non‐ruminant (0–3 weeks); transition phase (3–8 weeks), and rumination from 8 weeks (or weaning) onwards (Lane et al. 2002). During this process, the optimal growth and development of ruminal absorptive surface area, including the papillae, is crucial for the absorption and utilization of fermentation products, particularly VFA (Baldwin et al. 2004). Several factors have been identified as stimulants for rumen papillae development. These include the ingestion of dry feed (Greenwood et al. 1997), dietary supplementation with VFA, with butyrate being the most effective (O'Hara et al. 2018), and the presence of an epimural microbiota (Malmuthuge et al. 2012). Our study, which involved inoculation with rumen fermentation products (AUT) or microbiota (RFF and RFC) during the rumen development period, resulted in a lack of long‐term differences in the size of the different organs comprising the gastrointestinal tract (GIT). This lack of effects aligns with findings from a similar inoculation study in which lambs were slaughtered at 28 days of age (Yu et al. 2020). However, our study revealed that inoculation with fresh or autoclaved rumen fluid led to a modification of rumen papilla development. This resulted in wider rumen papillae (+42%) with a numerically higher surface area (+36%). Having papillae with a lower length‐to‐width ratio can be viewed as a positive dietary adaptation to absorb VFA. In this respect, it has been observed that high‐concentrate diets lead to an increased surface area of ruminal papillae and activity, increased thickness of the living stratum, and a premature transition of rumen epithelial cells to the stratum corneum (Steele et al. 2011).

Furthermore, our study revealed that this morphological adaptation was accompanied by molecular adaptations. In comparison to CTL animals, the inoculated animals (AUT, RFF and RFC) exhibited higher expression of several rumen epithelial genes. These included the Cyclin A gene (+63%), which is related to epithelial growth proliferation. Cyclin A‐CDK2 complexes drive chromosome duplication through the phosphorylation of key DNA replication factors (Norbury and Nurse 1992). Additionally, the MCT1 gene showed a remarkable increase (+421%). This particular gene is located at the basal side of the ruminal epithelium and contributes to the transport of protons from the ruminal epithelium into the blood, playing a key role in VFA absorption (Malhi et al. 2013). The HMGCL gene also displayed elevated expression (+164%), and it is involved in VFA metabolism, serving as the rate‐limiting enzyme in ketogenesis in the ruminal epithelium, leading to the production of β‐hydroxybutyrate (Naeem et al. 2012). Moreover, the inoculation led to numerical increases in complementary genes related to epithelial growth (IGFR1), cell proliferation (Cyclin D1 and CDK2), VFA absorption (PAT1) and VFA metabolism (BDH1 and HMGCS1). Increased proliferation of rumen epithelium and VFA absorption have also been observed in young lambs fed starter feed instead of milk feeding (Sun et al. 2018). These findings suggest that the rumen epithelium of the inoculated animals could exhibit superior abilities for VFA absorption and metabolism. Interestingly, the positive effects on gene expression were particularly prominent in the rumen epithelium of RFF animals, potentially explaining their higher blood β‐hydroxybutyrate and glucose concentrations and superior anion gap across treatments. A high anion gap indicates a substantial blood load of organic acids from the GIT (Gentile et al. 2004) and is often associated with an increased level of blood HCO_3_^−^ a compensatory mechanism to maintain blood pH within a physiological range, as observed in our study.

Hindgut microbial fermentation plays a vital role in gut health and is highly influenced by the availability of fermentable substrates and the type of gut microbiota (Guilloteau et al. 2010). In our study, animals inoculated with rumen fluid exhibited higher levels of certain bacterial taxa compared to CTL animals, with *Ruminococcacea* and *Faecalibacterium* being the most abundant. Additionally, a more diverse and abundant methanogens community was observed in inoculated animals. However, it is challenging to determine whether these differences can be attributed to a direct effect associated with a modification of the hindgut colonization process or if they result from an indirect effect mediated by the type of substrate entering the cecum (Yáñez‐Ruiz et al. 2015). Our findings suggest that this later hypothesis is unlikely because no differences were noted in the hindgut fermentation pattern, including VFA and pH. Moreover, the bacterial community was similar across treatments in terms of community structure, concentration, and diversity. This suggests that the diet was predominantly fermented in the rumen. However, the inoculated animals displayed a lower concentration of anaerobic fungi and lower expression of genes in the cecal epithelium associated with VFA absorption (MCT1 and PAT1) and VFA metabolism (BDH1). These findings imply a reduced availability of fermentable substrates and nutrient absorption in the hindgut for the inoculated animals, possibly as a result of a more efficient nutrient digestion in upper sections of the GIT.

## 6 Conclusions

This study clearly demonstrated that the inoculation of young ruminants with fresh rumen fluid from adult counterparts resulted in a significantly greater overall rumen microbial complexity. This was characterized by higher diversity among bacteria and methanogens, along with the presence of a diverse protozoal community which persisted later in life. The increased rumen microbial complexity provided a higher resilience for adult animals facilitating a quicker adaptation to high‐concentrate diets, mostly due to the presence of rumen protozoa. As a result, inoculated animals experienced lower incidence of rumen acidosis and diarrhoea leading to higher BW gains during the diet adaptation process. Moreover, once adapted to the high‐concentrate diet, inoculated animals also had higher rumen VFA concentration, rumen papillae width and increased expression of genes involved in the cell proliferation (Cyclin 1), VFA absorption (MCCT1) and VFA metabolism (HMGCL) in the rumen epithelium suggesting positive effects on the energy uptake by the ruminant. Despite differences in the rumen and hindgut microbiota were noted across treatments, they had no impact in terms of feed digestibility, N utilization or microbial protein synthesis, possibly as a compensation mechanism. In conclusion, these findings suggest that promoting greater rumen microbial diversity is a desirable attribute to prevent digestive disorders during the adaptation process to high‐concentrate diets despite having minor effects once the animals are fully adapted to the diet.

## Author Contributions

**Alejandro Belanche:** conceptualization, validation, methodology, investigation, resources and formal analysis, data curation, software, supervision and writing original draft. **Juan Manuel Palma‐Hidalgo:** methodology and resources. **David R. Yáñez‐Ruiz:** conceptualization, funding acquisition and supervision. All authors read and approved the final version.

## Ethics Statement

The authors confirm that the ethical policies of the journal, as noted on the journal's author guidelines page, have been adhered to and the appropriate ethical review committee approval has been received. The authors confirm that animal procedures were carried out in accordance to the EU standards and National guidelines (RD53/2013) and experimental protocols were approved by the EEZ‐CSIC Ethical Committee for Animal Research and regional government (09/03/2017).

## Conflicts of Interest

The authors declare no conflicts of interest.

## Supporting information


**Table S1:** Primers used for quantitative PCR and Next Generation Sequencing. **Table S2:** Rumen fermentation and microbial composition of the inocula. **Table S3:** Spearman's correlations (ρ > 0.4, *P* < 0.001) between the rumen microbes and productive data. **Figure S1:** Principal Coordinate Analysis illustrating the structure of the rumen multi‐kingdom microbiota in 7‐mongh‐old goats at 21 days after an abrupt dietary shift from a forage diet to a high‐concentrate diet.

## Data Availability

Raw sequences reads were deposited at the European Nucleotide Archive repository (PRJEB63607).
