# Supplementary material for: Increasing Rumen Microbial Diversity in Goats Favours the Adaptation to High‐Concentrate Diets With Minor Effects on Feed Utilization
Source: J Anim Physiol Anim Nutr (Berl). 2025 Oct 28;110(1):148–68. doi: 10.1111/jpn.70024 (PMC12824430; doi:10.1111/jpn.70024)
Supplement: Supplementary file 1 — Table S1: Primers used for quantitative PCR and Next Generation Sequencing. Table S2: Rumen fermentation and microbial composition of the inocula. Table S3: Spearman's correlations (ρ > 0.4, P < 0.001) between the rumen microbes and productive data. Figure S1: Principal Coordinate Analysis illustrating the structure of the rumen multi‐kingdom microbiota in 7‐mongh‐old goats at 21 days after an abrupt dietary shift from a forage diet to a high‐concentrate diet. [file JPN-110-148-s001.docx]

**SUPPORTING INFORMATION**

**Increasing rumen microbial diversity in goats favours the adaptation to high-concentrate diets with minor effects on feed utilization**

Alejandro Belanche^*^, Juan Manuel Palma-Hidalgo, David R. Yáñez-Ruiz

Correspondence: belanche@unizar.es

**Supporting Table S1.** Primers used for quantitative PCR and Next Generation Sequencing.

| Target | Ref. | Gene ID | Forward Primer | Reverse Primer | Amplicon (bp) |
| --- | --- | --- | --- | --- | --- |
| Quantitative PCR |  |  |  |  |  |
| Total bacteria | (Maeda et al., 2003) | 1048F/1175R | GTGSTGCAYGGYTGTCGTCA | ACGTCRTCCMCACCTTCCTC | 150 |
| Methanogens | (Denman et al., 2007) | qmcrA-F/qmcrA-R | TTCGGTGGATCDCARAGRGC | GBARGTCGWAWCCGTAGAATCC | 140 |
| Protozoa | (Sylvester et al., 2004) | P-SSU 316f/539r | GCTTTCGWTGGTAGTGTATT | CTTGCCCTCYAATCGTWCT | 223 |
| Anaerobic fungi | (Denman and McSweeney, 2006) | none | GAGGAAGTAAAAGTCGTAACAAGGTTTC | CAAATTCACAAAGGGTAGGATGATT | 120 |
| Sequencing |  |  |  |  |  |
| Bacteria | (Sim et al., 2012) | V3_F357/V5_926 | CCTACGGGAGGCAGCAG | CCGTCAATTCMTTTRAGT | 570 |
| Methanogens | (Gantner et al., 2011) | Arch349F/Arch806R | GYGCASCAGKCGMGAAW | GGACTACVSGGGTATCTAAT | 457 |
| Protozoa | (Hadziavdic et al., 2014) | F566Euk/R1200Euk | CAGCAGCCGCGGTAATTCC | CCCGTGTTGAGTCAAATTAAGC | 660+var. |
| Anaerobic fungi | (Op De Beeck et al., 2014) | ITS3/ITS4 | GCATCGATGAAGAACGCAGC | TCCTCCGCTTATTGATATGC | 356+var. |
| Gene expression |  |  |  |  |  |
| *MCT1* | (Laarman et al., 2012) | NM_001037319 | ATCTACGCGGGATTCTTTGGAT | AAGGTCCATCAGCGTTTCAAAC | 72 |
| *PAT1* | (Sun et al., 2018) | NW_011942300.1 | TGGACTGCCCCCCGTGTAT | CCAGAGCGGCTGCTTAGAT | 391 |
| *BHD1* | (Sun et al., 2018) | NW_014639010.1 | GAGAAGGAAACGGCGGTAG | AAAAGGCAGAATGGTCAGG | 169 |
| *HMGCS1* | (Sun et al., 2018) | NW_014639025.1 | ACAGAAGAACCTACGCTCG | CTCCCCGTTACTGATGACA | 172 |
| *HMGCL* | (Sun et al., 2018) | NW_011942261.1 | TGGGGACTGTGTGGGAATA | CAGCAGCAAGTTGTGGAGA | 312 |
| *Cyclin D1* | (Sun et al., 2018) | NC_019478.2 | CCTGCCGTCCATGCGGAA | GAACTTCACATCTGTGGCAC | 403 |
| *Cyclin A* | (Sun et al., 2018) | NC_019478.2 | CTCTCCTATCACCGCCTGAC | CTTTGGGGTCCAAGTTCTGC | 144 |
| *CDK2* | (Sun et al., 2018) | NM_001142509.1 | CCTAGCTTTCTGCCACTCTCAT | TCACCACCTCGTGGGTATAAGT | 153 |
| *IGF1* | (Sun et al., 2018) | EF012204.1 | GCTCTCAACATCTCCCATCTCC | CCCATTGCTTCTGAAGTGCAAA | 94 |
| *IGF1R* | (Sun et al., 2018) | NC_019475.2 | AGAAGATCACCATGAGCCGC | TCACCGTCTTAATGGCCACC | 120 |
| *BCL-2* | (Sun et al., 2018) | XM_012103831.2 | GTGGATGACCGAGTACCTGAAC | CTTCACTTATGGCCCAGATAGG | 197 |
| *BAX* | (Sun et al., 2018) | XM_015100640.1 | TGTCCTCCCCCAGAGATCAG | GGGCCCTAGAGGAGAAAGGA | 97 |
| Caspase-3 | (Sun et al., 2018) | XM_015104559.1 | CAGCTACCTCAAACACAGTTGG | TGATACAGTGGCATACCCACAT | 203 |
| *β-actin* | (Charavaryamath et al., 2011) | AF191490 | CTAGGCACCAGGGCGTAATG | CCACACGGAGCTCGTTGTAG | 177 |

**Supporting Table S2.** Rumen fermentation and microbial composition of the inocula.

| **Inoculum^1^** | **AUT** | **RFF** | **RFC** | **s.e.d.** | **P-value** |
| --- | --- | --- | --- | --- | --- |
| Fermentation products | | |  |  |  |
| pH | 6.11^b^ | 6.38^a^ | 5.79^c^ | 0.095 | 0.002 |
| Lactate (mM) | 1.86^a^ | 0.55^b^ | 0.85^b^ | 0.328 | 0.017 |
| Ammonia-N (mg/dL) | 8.64 | 7.27 | 10.0 | 1.095 | 0.117 |
| Total VFA (mM) | 120^ab^ | 103^b^ | 134^a^ | 9.990 | 0.055 |
| Acetate (%) | 63.0^b^ | 70.1^a^ | 55.5^c^ | 1.339 | <0.001 |
| Propionate (%) | 23.6^b^ | 18.2^c^ | 29.0^a^ | 1.711 | <0.001 |
| Butyrate (%) | 10.3^b^ | 9.49^c^ | 11.8^a^ | 0.746 | 0.004 |
| Bacterial community | |  |  |  |  |
| Concentration (log10 copies/l) | | 9.96 | 11.8 | 0.884 | 0.081 |
| Richness |  | 502 | 396 | 30.30 | 0.025 |
| Shannon index | | 4.50 | 4.21 | 0.170 | 0.154 |
| Abundance (%) | |  |  |  |  |
| p_Actinobacteriota | | 0 | 0.26 | 0.150 | 0.158 |
| p_Bacteroidota | | 71.5 | 59.1 | 3.060 | 0.015 |
| p_Cyanobacteria | | 0.66 | 0.15 | 0.118 | 0.012 |
| p_Elusimicrobiota | | 0.31 | 0.21 | 0.165 | 0.577 |
| p_Fibrobacterota | | 0.70 | 1.60 | 0.811 | 0.326 |
| p_Firmicutes | | 24.2 | 34.6 | 2.990 | 0.025 |
| p_Proteobacteria | | 2.06 | 0.83 | 0.669 | 0.140 |
| p_Spirochaetota | | 0.21 | 2.31 | 0.930 | 0.087 |
| p_Synergistota | | 0.09 | 0.10 | 0.048 | 0.812 |
| p_Verrucomicrobiota | | 0.07 | 0.04 | 0.026 | 0.221 |
| Firmicutes / Bacteroidota | | 0.34 | 0.59 | 0.070 | 0.023 |
| Methanogens community | | |  |  |  |
| Concentration (log10 copies/ml) | | 6.32 | 7.65 | 1.213 | 0.314 |
| Richness |  | 20.5 | 14.5 | 1.683 | 0.025 |
| Shannon index | | 1.85 | 1.75 | 0.240 | 0.688 |
| Abundance (%) | |  |  |  |  |
| f_Methanobacteriaceae | | 35.8 | 55.2 | 33.30 | 0.043 |
| f_Methanomassiliicoccaceae | | 62.6 | 44.8 | 38.00 | 0.079 |
| f_Methanomicrobiaceae | | 1.61 | 0 | 4.620 | 0.158 |
| *g_Methanobrevibacter* | | 28.7 | 55.2 | 23.10 | 0.005 |
| *g_Methanosphaera* | | 7.12 | 0 | 15.88 | 0.089 |
| Protozoal community | |  |  |  |  |
| Concentration (log10 copies/ml) | | 6.6 | 8.7 | 0.493 | 0.005 |
| Richness |  | 25.0 | 27.0 | 2.890 | 0.527 |
| Shannon index | | 2.24 | 2.46 | 0.187 | 0.297 |
| Abundance (%) | |  |  |  |  |
| *g_Entodinium* | | 48.8 | 49.3 | 0.102 | 0.863 |
| *g_Ophryoscolex* | | 3.19 | 9.02 | 0.737 | 0.126 |
| *g_Diplodinium* | | 0.01 | 0.01 | 0.123 | 0.999 |
| *g_Polyplastron* | | 0.66 | 2.84 | 0.548 | 0.087 |
| *g_Enoploplastron* | | 0.83 | 0 | 0.101 | <0.001 |
| *g_Isotricha* |  | 18.6 | 22.7 | 0.215 | 0.969 |
| *g_Dasytricha* |  | 25.5 | 9.4 | 0.046 | <0.001 |
| *g_*Unclassified | | 2.31 | 6.7 | 0.481 | 0.121 |
| Anaerobic fungal community | | |  |  |  |
| Concentration (log10 copies/ml) | | 6.76 | 7.55 | 0.125 | <0.001 |
| Richness |  | 12.0 | 15.0 | 2.380 | 0.276 |
| Shannon index | | 1.32 | 1.63 | 0.527 | 0.501 |
| Abundance (%) | |  |  |  |  |
| *g_Caecomyces* | | 63.6 | 9.22 | 0.655 | 0.064 |
| *g_Piromyces* |  | 0.0 | 50.0 | 0.006 | <0.001 |
| *g_Neocallimastigaceae* | | 36.4 | 40.8 | 0.107 | 0.621 |

Treatments: Autoclaved rumen fluid (AUT), fresh rumen fluid from adult goats adapted to forage-rich (RFF) or concentrate-rich diet (RFC).

**Supporting Table S3.** Spearman´s correlations (*ρ*>0.4, *P*<0.001) between the rumen microbes and productive data.

| **Taxa** | **pH** | **NH_3_** | **Lact.** | **VFA** | **Ace.** | **Prop.** | **But.** | **DMI** | **OMd** | **Nd** | **NDFd** | **PD_C** | **EMPS** |
| --- | --- | --- | --- | --- | --- | --- | --- | --- | --- | --- | --- | --- | --- |
| Fibrobacter/Bacteroidata |  |  |  |  |  |  |  |  | -0.45 | -0.47 |  |  |  |
| p_Actinobacteriota | -0.68 |  |  | 0.43 |  |  |  | -0.50 |  |  |  |  |  |
| p_Elusimicrobiota |  |  |  |  |  |  |  | -0.45 | -0.43 |  |  |  |  |
| p_Spirochaetota |  |  | -0.55 |  |  | -0.44 |  |  | 0.57 | 0.46 |  | -0.43 | -0.47 |
| p_Synergistota |  |  | 0.47 |  |  |  | -0.42 |  |  |  | -0.56 |  |  |
| p_Other_phylum |  |  |  |  | 0.46 | -0.44 |  | -0.47 |  |  | 0.45 | -0.65 |  |
| f_Atopobiaceae | -0.60 |  |  | 0.46 |  |  |  | -0.49 |  |  |  |  |  |
| f_Barnesiellaceae |  |  |  |  |  |  | 0.47 |  |  |  |  |  |  |
| f_Prevotellaceae |  | 0.56 |  |  |  |  |  |  |  |  |  |  |  |
| f_Acidaminococcaceae | 0.44 |  | -0.43 |  | 0.49 | -0.53 |  |  |  |  |  |  |  |
| f_Anaerovoracaceae | 0.48 | 0.64 |  |  |  | -0.53 |  |  |  |  |  |  |  |
| f_Erysipelatoclostridiaceae |  |  |  | 0.54 |  |  |  | -0.53 |  |  |  |  |  |
| f_Erysipelotrichaceae |  |  |  | 0.44 |  |  | 0.47 |  |  |  |  |  |  |
| f_Hungateiclostridiaceae |  | 0.53 |  |  |  |  |  |  |  |  |  |  |  |
| f_Lachnospiraceae |  |  |  |  |  | -0.49 | 0.68 |  |  |  |  |  |  |
| f_Oscillospiraceae | -0.54 |  |  | 0.49 |  |  | 0.47 |  |  |  |  |  |  |
| f_Selenomonadaceae |  | 0.47 |  |  |  |  |  |  |  |  |  |  |  |
| g_Bacteroidales_RF16_group |  |  | 0.45 |  |  |  |  |  |  |  |  |  |  |
| g_Bacteroidales_UCG-001 |  |  |  | 0.43 |  |  |  |  |  |  |  |  |  |
| g_F082 |  | 0.45 |  |  |  | -0.44 |  |  |  |  |  |  |  |
| g_p-2534-18B5_gut_group | -0.58 |  |  |  |  |  |  |  |  |  |  |  |  |
| g_Prevotella | 0.50 | 0.46 |  |  |  |  |  |  |  |  |  |  |  |
| g_Prevotellaceae_Ga6A1_group |  |  |  |  |  |  | -0.53 |  |  |  |  |  |  |
| g_Prevotellaceae_UCG-001 |  |  |  |  |  |  |  |  |  |  | -0.47 |  |  |
| g_Prevotellaceae_YAB2003_group | -0.49 |  |  |  |  |  | 0.45 |  |  |  | 0.43 |  |  |
| g_Rikenellaceae_RC9_gut_group |  |  |  |  | 0.47 |  |  | -0.55 |  |  |  |  |  |
| g_Elusimicrobium |  |  |  |  |  |  |  | -0.44 | -0.47 |  |  |  |  |
| g_[Eubacterium]_coprostanoligenes_group |  |  |  |  |  |  | 0.48 |  |  |  |  |  |  |
| g_Succiniclasticum | 0.43 |  |  |  | 0.47 | -0.58 |  |  |  |  |  |  |  |
| g_Anaerovorax |  | 0.74 |  |  |  | -0.46 |  |  |  |  |  |  |  |
| g_Family_XIII_AD3011_group |  | 0.63 |  |  |  |  |  |  |  |  |  |  |  |
| g_Clostridium_sensu_stricto_1 |  |  |  |  |  |  | 0.47 |  |  |  |  |  |  |
| g_UCG-004 |  |  |  | 0.54 |  |  |  | -0.56 |  |  |  |  |  |
| g_Saccharofermentans |  | 0.53 |  |  |  |  |  |  |  |  |  |  |  |
| g_Izemoplasmatales |  | 0.43 |  |  |  | -0.47 | 0.46 |  |  |  |  |  |  |
| g_Acetitomaculum | 0.42 | 0.59 |  |  |  |  |  |  |  |  |  |  |  |
| g_Anaerostipes | -0.48 |  |  |  |  |  |  |  |  |  |  |  |  |
| g_Butyrivibrio | 0.47 |  |  |  |  |  |  |  |  |  |  |  |  |
| g_Lachnospiraceae_NK3A20_group |  | 0.67 |  |  | 0.44 | -0.57 | 0.40 |  |  |  |  |  |  |
| g_Lachnospiraceae_NK4A136_group |  |  |  | 0.48 |  |  |  |  |  |  |  |  |  |
| g_Lachnospiraceae_XPB1014_group | 0.59 |  |  |  | 0.50 |  |  |  |  |  |  |  |  |
| g_Moryella |  | 0.47 |  | 0.49 |  |  |  |  |  |  |  |  |  |
| g_Oribacterium |  | 0.54 |  |  |  |  |  |  |  |  |  |  |  |
| g_NK4A214_group |  |  |  | 0.49 |  | -0.43 |  |  |  |  |  |  |  |
| g_UCG-005 | -0.47 |  |  |  |  |  |  |  |  |  |  |  |  |
| g_Terrisporobacter | -0.43 |  |  |  |  |  |  |  |  |  |  |  |  |
| g_CAG-352 | -0.49 |  |  | 0.67 |  |  |  |  |  |  |  |  |  |
| g_Candidatus_Soleaferrea |  | 0.47 |  |  |  |  |  |  |  |  |  |  |  |
| g_Faecalibacterium |  |  |  | 0.44 |  |  |  |  |  |  |  |  |  |
| g_Anaerovibrio | 0.40 | 0.55 |  |  |  |  |  |  |  |  |  |  |  |
| g_Quinella |  |  | 0.76 |  | -0.52 | 0.53 |  |  |  | -0.42 | -0.62 | 0.50 |  |
| g_Selenomonas |  |  |  |  |  |  |  |  |  |  |  |  | 0.48 |
| g_Veillonellaceae_UCG-001 |  | 0.43 |  |  |  |  |  |  |  |  |  |  |  |
| g_Succinivibrio |  |  |  |  | -0.45 |  |  |  |  |  |  |  |  |
| g_Succinivibrionaceae_UCG-002 | -0.42 |  |  | 0.62 |  |  | 0.42 |  |  |  |  |  |  |
| g_Sphaerochaeta |  | -0.46 |  |  |  |  |  |  |  |  |  |  |  |
| g_Treponema |  |  | -0.49 |  |  |  |  |  | 0.48 |  |  |  | -0.46 |
| g_Fretibacterium |  |  | 0.54 |  |  |  | -0.47 |  |  |  |  |  |  |
| s_Olsenella_umbonata | -0.68 |  |  | 0.53 |  |  |  | -0.60 |  |  |  |  |  |
| s_Bacteroidales_bacterium |  |  |  |  |  |  |  | -0.49 |  |  |  |  |  |
| s_Fibrobacter_succinogenes |  |  | 0.50 |  |  |  |  |  |  |  |  |  |  |
| s_Lachnospiraceae_bacterium |  | 0.44 |  |  |  |  |  | -0.45 |  |  |  |  |  |
| s_bacterium_ND2018 |  | 0.54 |  | 0.53 |  | -0.48 | 0.50 | -0.57 |  |  |  |  |  |
| s_bacterium_YRD2003 |  |  | 0.46 |  | -0.49 | 0.49 |  |  | -0.44 |  |  |  |  |
| s_Ruminococcus_flavefaciens |  |  |  |  |  |  |  |  | -0.43 |  |  |  |  |
| s_Anaerovibrio_sp. |  |  |  | 0.47 |  |  |  |  |  |  |  |  |  |
| s_Ruminobacter_amylophilus | 0.47 |  |  |  |  |  |  |  |  | 0.44 |  |  |  |
| s_Treponema_ruminis |  | -0.63 |  |  |  |  |  |  |  |  |  |  |  |
| Methanogens concentration |  | 0.54 |  |  |  | -0.48 |  |  |  |  |  |  |  |
| Methanogens richness |  |  |  |  |  | -0.55 | 0.47 |  |  |  |  |  |  |
| g_Group10 |  | -0.43 |  |  |  |  |  |  |  | -0.56 |  |  |  |
| g_Group11 |  | -0.69 |  |  |  |  |  |  |  |  |  |  |  |
| s_ Methanobrevibacter_gottschalkii_clade |  |  |  |  | 0.42 | -0.45 |  |  |  |  |  |  |  |
| s_ Methanobrevibacter_oralis |  |  |  |  |  |  |  |  |  |  | -0.46 | 0.45 |  |
| s_ Group11_sp ISO4-G11 |  | -0.46 |  |  |  |  |  |  |  |  |  |  |  |
| s_ Group12_sp ISO4-H5 |  |  |  |  |  |  |  |  |  |  | 0.49 |  |  |
| s_ Group9_sp ISO4-G1 |  | -0.50 |  |  |  |  |  |  |  |  |  |  |  |
| Protozoal concentration |  | 0.73 |  |  |  | -0.58 | 0.53 |  |  |  |  |  |  |
| Protozoal Richness |  | 0.44 |  |  |  |  |  |  |  |  |  |  |  |
| g_ Entodinium |  | 0.47 | 0.47 |  |  |  |  |  |  |  |  |  |  |
| g_ Ophryoscolex |  | 0.62 | -0.48 |  | 0.45 | -0.67 | 0.57 | -0.44 |  |  |  |  |  |
| g_ Polyplastron |  | 0.56 |  |  |  |  |  |  |  | 0.42 |  |  |  |
| s_ Isotricha_prostoma |  |  |  | 0.46 |  |  |  |  |  |  |  |  |  |
| s_ Isotricha_intestinalis |  |  |  | 0.42 |  |  |  |  |  |  |  |  |  |
| Anaerobic fungal concentration |  |  |  |  | 0.63 | -0.59 |  |  |  |  |  | -0.42 |  |
| Anaerobic fungal Richness |  |  |  |  |  |  |  |  |  |  | 0.43 |  |  |
| g_Anaeromyces |  |  |  |  |  |  |  |  | 0.45 |  | 0.43 |  |  |

Abbreviations: Ace, Acetate; Pro, Propionate; But, Butyrate; Lact, Lactate; PD_, Purine derivatives: creatinine ratio; d, digestibility; EMPS, Efficiency of microbial protein synthesis.


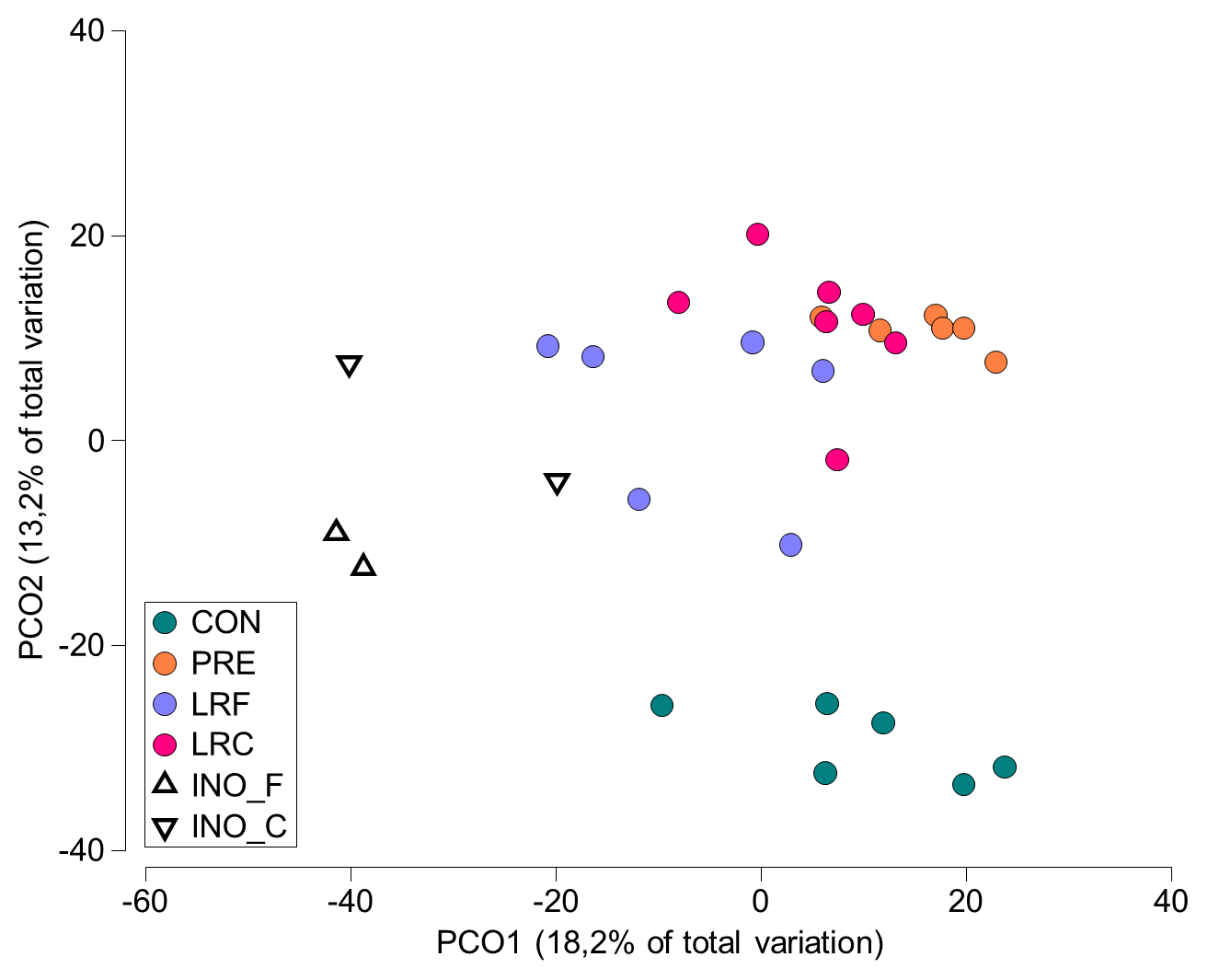
**Supporting Fig. S1.** Principal Coordinate Analysis illustrating the structure of the rumen multi-kingdom microbiota in 7-mongh-old goats at 21 days after an abrupt dietary shift from a forage diet to a high-concentrate diet. Goats received early-life daily inoculations during their first 10 weeks of age with autoclaved rumen fluid (AUT), rumen fluid from adult animals fed a forage diet (RFF) or concentrate diet (RFC), or no inoculation (CTL). Rumen fluid used as microbial inocula adapted to forage (INO-F) or high-concentrated diet (INO_C) is also provided.

**REFERENCES**

Op De Beeck, M., B. Lievens, P. Busschaert, S. Declerck, J. Vangronsveld, and J. V. Colpaert. 2014. Comparison and Validation of Some ITS Primer Pairs Useful for Fungal Metabarcoding Studies. PLoS One 9:e97629. doi:10.1371/JOURNAL.PONE.0097629.

Charavaryamath, C., P. Fries, S. Gomis, C. Bell, K. Doig, L.L. Guan, A. Potter, S. Napper, and P.J. Griebel. 2011. Mucosal changes in a long-term bovine intestinal segment model following removal of ingesta and microflora. https://doi.org/10.4161/gmic.2.3.16483 2:134–144. doi:10.4161/GMIC.2.3.16483.

Denman, S.E., and C.S. McSweeney. 2006. Development of a real-time PCR assay for monitoring anaerobic fungal and cellulolytic bacterial populations within the rumen.. FEMS Microbiol. Ecol. 58:572–582.

Denman, S.E., N. Tomkins, and C.S. McSweeney. 2007. Quantitation and diversity analysis of ruminal methanogenic populations in response to the antimethanogenic compound bromochloromethane. FEMS Microbiol. Ecol. 62:313–322. doi:10.1111/j.1574-6941.2007.00394.x.

Gantner, S., A.F. Andersson, L. Alonso-Sáez, and S. Bertilsson. 2011. Novel primers for 16S rRNA-based archaeal community analyses in environmental samples. J. Microbiol. Methods 84:12–18. doi:10.1016/j.mimet.2010.10.001.

Hadziavdic, K., K. Lekang, A. Lanzen, I. Jonassen, E.M. Thompson, and C. Troedsson. 2014. Characterization of the 18s rRNA gene for designing universal eukaryote specific primers. PLoS One 9:e87624. doi:10.1371/journal.pone.0087624.

Laarman, A.H., A.L. Ruiz-Sanchez, T. Sugino, L.L. Guan, and M. Oba. 2012. Effects of feeding a calf starter on molecular adaptations in the ruminal epithelium and liver of Holstein dairy calves. J. Dairy Sci. 95:2585–2594. doi:10.3168/jds.2011-4788.

Maeda, H., C. Fujimoto, Y. Haruki, T. Maeda, S. Kokeguchi, M. Petelin, H. Arai, I. Tanimoto, F. Nishimura, and S. Takashiba. 2003. Quantitative real-time PCR using TaqMan and SYBR Green for Actinobacillus actinomycetemcomitans , Porphyromonas gingivalis , Prevotella intermedia , tetQ gene and total bacteria. FEMS Immunol Med Microbiol 39:81 – 86.

Sim, K., M.J. Cox, H. Wopereis, R. Martin, J. Knol, M.S. Li, W.O.C.M. Cookson, M.F. Moffatt, and J.S. Kroll. 2012. Improved detection of bifidobacteria with optimised 16S rRNA-gene based pyrosequencing. PLoS One 7:e32543. doi:10.1371/journal.pone.0032543.

Sun, D.M., S.Y. Mao, W.Y. Zhu, and J.H. Liu. 2018. Effect of starter diet supplementation on rumen epithelial morphology and expression of genes involved in cell proliferation and metabolism in pre-weaned lambs. animal 12:2274–2283. doi:10.1017/S1751731118000290.

Sylvester, J.T., S.K.R. Karnati, Z.T. Yu, M. Morrison, and J.L. Firkins. 2004. Development of an assay to quantify rumen ciliate protozoal biomass in cows using real-time PCR.. J. Nutr. 134:3378–3384.
